# Supplementary material for: Challenges associated with e-cigarette use by people in custody in Scottish prisons: a qualitative interview study with prison staff
Source: BMJ Open. 2022 Feb 18;12(2):e051009. doi: 10.1136/bmjopen-2021-051009 (PMC8860047; doi:10.1136/bmjopen-2021-051009)
Supplement: Author's manuscript [file bmjopen-2021-051009.draft_revisions.pdf]

BMJ Open

BMJ Open is committed to open peer review. As part of this commitment we make the peer review history of every article we publish publicly available.

When an article is published we post the peer reviewers' comments and the authors' responses online. We also post the versions of the paper that were used during peer review. These are the versions that the peer review comments apply to.

The versions of the paper that follow are the versions that were submitted during the peer review process. They are not the versions of record or the final published versions. They should not be cited or distributed as the published version of this manuscript.

BMJ Open is an open access journal and the full, final, typeset and author-corrected version of record of the manuscript is available on our site with no access controls, subscription charges or pay-per-view fees (http://bmjopen.bmj.com).

If you have any questions on BMJ Open's open peer review process please email info.bmjopen@bmj.com

BMJ Open

The challenges associated with e-cigarette use by people in custody in Scottish prisons: a qualitative interview study with prison staff

Journal:	*BMJ Open*
Manuscript ID	bmjopen-2021-051009
Article Type:	Original research
Date Submitted by the Author:	08-Mar-2021
Complete List of Authors:	O'Donnell, R; University of Stirling, Institute of Social Marketing and Health<br>Brown, Ashley; University of Stirling Institute for Social Marketing<br>Eadie, Douglas; Institute for Social Marketing, School of Health Sciences<br>Mitchell, Danielle; Institute for Social Marketing and Health, University of Stirling, Faculty of Health Sciences and Sport<br>Bauld, Linda; University of Edinburgh, Usher Institute and UK Centre for Tobacco and Alcohol Studies, College of Medicine and Veterinary Medicine<br>Demou, Evangelia; University of Glasgow, MRC/CSO Social & Public Health Sciences Unit<br>Purves, Richard; University of Stirling, Institute for Social Marketing and Health<br>Sweeting , Helen ; University of Glasgow, MRC/CSO Social & Public Health Sciences Unit<br>Hunt, Kate; University of Stirling Institute for Social Marketing and Health, Institute for Social Marketing and Health
Keywords:	PUBLIC HEALTH, QUALITATIVE RESEARCH, OCCUPATIONAL & INDUSTRIAL MEDICINE

SCHOLARONE™
Manuscripts

I, the Submitting Author has the right to grant and does grant on behalf of all authors of the Work (as defined in the below author licence), an exclusive licence and/or a non-exclusive licence for contributions from authors who are: i) UK Crown employees; ii) where BMJ has agreed a CC-BY licence shall apply, and/or iii) in accordance with the terms applicable for US Federal Government officers or employees acting as part of their official duties; on a worldwide, perpetual, irrevocable, royalty-free basis to BMJ Publishing Group Ltd ("BMJ") its licensees and where the relevant Journal is co-owned by BMJ to the co-owners of the Journal, to publish the Work in this journal and any other BMJ products and to exploit all rights, as set out in our licence.

The Submitting Author accepts and understands that any supply made under these terms is made by BMJ to the Submitting Author unless you are acting as an employee on behalf of your employer or a postgraduate student of an affiliated institution which is paying any applicable article publishing charge ("APC") for Open Access articles. Where the Submitting Author wishes to make the Work available on an Open Access basis (and intends to pay the relevant APC), the terms of reuse of such Open Access shall be governed by a Creative Commons licence – details of these licences and which Creative Commons licence will apply to this Work are set out in our licence referred to above.

Other than as permitted in any relevant BMJ Author's Self Archiving Policies, I confirm this Work has not been accepted for publication elsewhere, is not being considered for publication elsewhere and does not duplicate material already published. I confirm all authors consent to publication of this Work and authorise the granting of this licence.

For peer review only

The challenges associated with e-cigarette use by people in custody in Scottish prisons: a qualitative interview study with prison staff

Rachel O'Donnell[1*], Ashley Brown[1*], Douglas Eadie[1], Danielle Mitchell[1], Linda Bauld[2], Evangelia Demou[3], Richard Purves[1], Helen Sweeting[3] and Kate Hunt[1]

1. Institute for Social Marketing and Health, Faculty of Health Sciences and Sport, University of Stirling, Pathfoot Building, Stirling, FK9 4LA.

2. Usher Institute and SPECTRUM Consortium, College of Medicine and Veterinary Medicine, University of Edinburgh.

3. MRC/CSO Social and Public Health Sciences Unit, Berkeley Square, 99 Berkeley Street, Glasgow, G3 7HR.

* RO and AB are joint first authors and contributed equally to this work.

Corresponding author: Kate Hunt kate.hunt@stir.ac.uk

Word Count: 3696/4000 words

Keywords: e-cigarettes, vaping, prisoner health, staff, workplace, qualitative

For peer review only - http://bmjopen.bmj.com/site/about/guidelines.xhtml

ABSTRACT

Introduction: Little is known about the perspectives of staff working in prisons where e-cigarettes are permitted. Scotland now permits people in custody (PiC), but not staff/visitors to use e-cigarettes, following implementation of smokefree prisons policy in 2018. Previous studies, conducted before and immediately after the introduction of e-cigarettes in Scottish prisons, have evidenced stakeholder support for their use by PiC. This study focuses on key challenges associated with e-cigarette use in prisons, using data collected from prison staff once this had been allowed for several months.

Methods: Sixteen qualitative interviews were conducted with prison staff from five prisons varying by population (sex, age and sentence length). Data were managed and analysed using the Framework approach.

Results: Whilst these staff confirmed strong support for the smokefree prison policy and reported some benefits of replacing tobacco with e-cigarettes, they also spoke of the challenges e-cigarettes pose. These included: workplace e-cigarette vapour exposures; perceptions that e-cigarettes provide a new, effective way for some PiC to take illegal drugs, particularly new psychoactive substances; organisational challenges relating to the value attached to e-cigarettes in prisons; and implications for long-term nicotine use and tobacco cessation. Staff anticipated difficulties in tightening restrictions on e-cigarette use by PiC given its scale and significance among this population.

Conclusions: Maximizing the benefits of e-cigarette use by PiC is likely to require multiple measures to support effective and safe use and e-cigarette reduction/cessation where desired. Findings are relevant to jurisdictions considering or planning changes in prison smoking or vaping policies.

For peer review only - http://bmjopen.bmj.com/site/about/guidelines.xhtml

Strengths and limitations of the study

- This qualitative study provides in-depth insights into prison staff experiences of the challenges associated with allowing e-cigarettes to be sold and used among people in custody in prisons with established smokefree policies.
- This study broadens understanding of risks and challenges of use of e-cigarettes in this distinct setting and corresponding measures that might minimise harms and maximise benefits.
- The study findings highlight the need for policies on e-cigarette use in prisons to balance the needs of people in custody who wish to use e-cigarettes against concerns about exposures to e-cigarette vapour at work; use of e-cigarettes for illicit drug taking among people in custody; the value and trading of e-cigarette products in prisons; and implications for long-term nicotine and tobacco use.
- Whilst some staff groups (e.g. female members of staff, current vapers) may not be adequately represented in the sample, the inclusion of staff from a diverse set of prisons provides confidence that the breadth of challenges associated with e-cigarettes in prisons are likely to be reflected.

INTRODUCTION

In anticipation of the November 2018 implementation of the country's smokefree prison policy, legislative changes occurred which enabled people in custody (PiC) in Scotland to purchase and use rechargeable e-cigarettes in rooms (cells) and outdoor spaces from September 2018. Prison staff and visitors remained prohibited from smoking, or using e-cigarettes (hereafter vaping) on prison property. Information on e-cigarette products sold in Scottish prisons is available elsewhere.[1] This development (following similar changes in

For peer review only - http://bmjopen.bmj.com/site/about/guidelines.xhtml

jurisdictions such as England and Wales, and the US [2, 3]) has potentially important implications for the health and safety of PiC and prison staff, and the operation of prison systems. There is very limited evidence on e-cigarettes in prisons, with no studies reporting on the perspectives of staff working in prisons where vaping is permitted among PiC, thus limiting understandings of its perceived benefits and risks in this distinct setting.

Current research evidence on e-cigarettes in prisons comes from two studies of tobacco and vaping in Scottish prisons (the Tobacco in Prisons study [TIPs] and the E-cigarettes in Prisons study). Evidence from TIPs, and responses to a public consultation on smokefree prisons (by the Scottish Prison Service), showed that e-cigarettes received broad support from stakeholders in anticipation of the introduction of smokefree prison policy. For example, TIPs surveys in May-June 2018 (after the smokefree policy announcement but before it was known e-cigarettes would be made available to PiC) and May-June 2019 (6-9 months after use of (rechargeable) e-cigarettes was first allowed and smokefree policy was implemented in Scottish prisons) both showed the majority of prison staff (~75%) and almost all PiC (~90%) agreed that ‘e-cigarettes should be available to help prisoners to stop smoking/manage without tobacco’.[4]

Complementary qualitative research suggests this broad support for e-cigarettes in Scottish prisons has been substantially influenced by perceptions that they are beneficial in helping PiC and staff to cope with the major operational change represented by the removal of tobacco (Brown 2021, submitted). Favourable perceptions of e-cigarettes in Scottish prisons also reflect broader evidence on their appeal and effectiveness for some current and ex-smokers (e.g. those who feel unwilling or unable to quit nicotine)[5, 6] and evidence on reduced harms to users and bystanders from vaping relative to smoking.[7]

For peer review only

However, important issues and challenges in relation to permitting PiC to vape were also voiced by prison staff in anticipation of their introduction [8] (and some PiC in anticipation of and again 6-10 months after their implementation [1, 8, 9]), as well as by commentators in other jurisdictions.[10, 11] These concerns relate to e-cigarette safety, implications for long-term tobacco and nicotine cessation, and risks associated with vaping related to unique features of prison environments. These include: rules around earnings and purchases that mean PiC are not always able to (formally) buy (enough of) the items they want or 'need'; operation of illicit prison economies in which any sought-after item can be traded or 'bought' using alternative currencies; and well established practices of PiC repurposing items, including for illicit purposes such as drug taking.[1] The benefits of e-cigarettes in prisons are well covered in our previous publications (including focus groups with staff before rules were changed to allow PiC to vape;[8, 12] interviews with PiC immediately after e-cigarettes were introduced [1] and again once new rules on e-cigarettes and smoking had bedded in [9]; and repeat cross sectional surveys of staff and PiC [4]), This paper therefore focuses on the *challenges* of e-cigarettes in prisons identified by prison staff, based on novel interview data collected after vaping in prisons had become well established (8-11 months after sales of rechargeable e-cigarettes first began in Scottish prisons and 6-9 months after smokefree rules were implemented). Together with our previous studies, the current findings will be relevant for prison systems (and institutions such as mental health inpatient services) seeking to implement, and maximise the benefits of, smokefree policies.

METHODS

Sample and recruitment

Data were collected between May-August 2019 from 16 Scottish Prison Service staff working in five (of Scotland's 15) prisons which varied by prison population (sex, age and

For peer review only - http://bmjopen.bmj.com/site/about/guidelines.xhtml

sentence length) and capacity. Participants were recruited through designated staff contacts who were asked to invite staff from a range of work roles (e.g. residential; admissions; health and safety; prison management), to enable the exploration of diverse views. Our contacts scheduled one-to-one meetings between researchers and staff who expressed interest in participation. Sample characteristics are detailed in Table 1.

Table 1: Participant Characteristics

**Work role**		**Number of years of service**	
Residential officer	8	1-10	2
Non-residential officer	6	11-20	6
Managerial	1	21-30	5
Missing	1	31-40	2
		Missing	1
**Sex**		**Age (years)**	
Male	13	18-30	2
Female	3	31-40	2
		41-50	3
		51-60	8
		Missing	1
**Smoking status**		**Vaping status**	
Non smoker (NS)	9	Non vaper (NV)	11
Ex smoker (ExS)	4	Ex vaper (ExV)	3
Current smoker (CS)	2	Current vaper (CV)	1
Missing	1	Missing	1

For peer review only - http://bmjopen.bmj.com/site/about/guidelines.xhtml

Data collection

Interviews (averaging 35 minutes) were conducted in a room which allowed the participant to speak freely. After providing information about the study and answering any questions, written or audio-recorded verbal consent was obtained from each participant by one of three interviewers (RO, AB, DE) before s/he commenced the interview. The topic guide covered: participant background, smoking and vaping history; views and experiences related to vaping in the prison context; and views on the positive and negative consequences of vaping by PiC for prison staff, the prison regime and PiC themselves. Question wording and order were varied as appropriate and participants were invited to raise any additional points they considered important.

Data analysis

Fifteen of the 16 interviews were audio-recorded and transcribed by a professional transcription agency; detailed notes were made in lieu of audio-recording of one interview on participant request. All interview content was de-identified, then analysed (by RO, AB, DM) using the Framework approach.[13] This approach is well suited to the analysis of in-depth interview data, as summarisation of data into a framework grid within the NVivo program facilitates analysis across cases without losing the wider context of each participant's account.[14] A thematic framework was developed (by AB, RO) to guide data management and analysis, based on deductive (considering the topic guide, relevant literature) and inductive (reading transcripts) techniques. In preparation for detailed analysis, data summaries were written in relevant cells of the framework grid (RO, DM, AB), incorporating hyperlinks to transcripts to facilitate data retrieval. RO reviewed all summaries to check data interpretation and consistency of approach. Data summaries were then used to identify high level themes (AB, RO) before further in-depth analysis was conducted (RO), focusing on data about challenges posed by e-cigarettes in prisons. RO and AB finalised themes based on

For peer review only - http://bmjopen.bmj.com/site/about/guidelines.xhtml

re-examining data and reflexive team discussions. Themes are presented below alongside illustrative quotes in Tables 2-4; each quote indicates the prison worked in and the participant's interview ID number, and smoking and vaping status.

Patient and public involvement

The study was designed to ensure that the views of prison staff could be heard at different stages of the process of implementing smoke-free prisons in Scotland. A Research Advisory Group which included representation from prison management and union members provided feedback on the overall design of the study, study materials, and early findings to inform data collection and interpretation. Staff within the prisons were involved in the dissemination of information about the study and recruitment to the study. Personnel involved in implementing and managing smoke-free policies were also involved in discussion of findings to establish recommendations for future policy-makers and implementers.

RESULTS

Staff perceptions of the smoke free prison policy and e-cigarette availability for PiC

Prior to reporting staff perspectives of the *challenges* of e-cigarettes in prisons, we briefly summarise, for context, their opinions on smokefree policy and beliefs about the benefits of e-cigarettes in prisons. Staff interviewed for this study voiced strong overall support for the smokefree prison policy and reported positive impacts of removing tobacco from prisons, including benefits for their own health and comfort while at work (Table 2-1) (see also [1, 8]). E-cigarettes were generally believed to have supported smokefree prison policy implementation (Table 2-2) and benefits for reducing tobacco-related harms among PiC and staff were sometimes discussed. However, whilst most participants acknowledged some benefits associated with the introduction of e-cigarettes in prisons, several drawbacks were

For peer review only - http://bmjopen.bmj.com/site/about/guidelines.xhtml

also discussed, leading some to reflect on whether smokefree prisons could have been achieved without allowing PiC to vape (Table 2, 3-4).

Table 2: Staff perceptions of the smoke-free prison policy and e-cigarette availability

1. "It's night and day in that the smell of tobacco's not there. You're not breathing it in. …it's not in your clothes. You're not bringing it home to your family. That to me is a big thing." *L4-NS-NV*
2. "The fact that the e-cigarettes gave an alternative to the cigarettes actually made the transition of the jail changing over to no smoking a lot easier…it was a massive help to us." *B1-NS-NV*
3 "…making jails smoke free, we could just say, right that's it [no nicotine substitute], and we would have had bother [from PiC]. But would we have got through that bother, and then not had the smokers [vapers]?" *C2-NS-NV*
4 "The vapes made the process from smoking tobacco to there not being tobacco, it made that an easy process because we gave the prisoners a new option, while we kind of said to ourselves…'Why not just take the hit [i.e. without introducing e-cigarettes] and see how it goes with no tobacco?' Would that not have been better? Especially because I don't think there's enough research into the e-cigarettes and the vaping as well. We don't know exactly what kind of consequences there are." *O3-NS-NV*

For peer review only

<u>Staff exposures to e-cigarette vapour</u>

Several participants expressed doubts about the safety of vaping for themselves as bystanders (Table3a-1), in many cases referring to the absence of long-term studies on exposure to e-cigarette vapour (Table 3a-2). Concerns about exposure to e-cigarette vapour whilst at work were discussed in the context of very widespread vaping among PiC (Table 3a-3). In this context, some staff reported practical challenges in avoiding exposures to e-cigarette vapour when carrying out tasks such as cell searches (Table 3a-4). One participant wondered

For peer review only - http://bmjopen.bmj.com/site/about/guidelines.xhtml

whether the persistent cough she had started to experience was linked to workplace exposure to e-cigarette vapour.

Table 3: Staff exposures to e-cigarette vapour, and misuse of e-cigarettes among PiC to take illegal drugs

3a) Staff exposures to e-cigarette vapour

1. "I wonder what the harm is of passive smoking with the vapes. I know we've taken away a lot of chemicals and tar and things that were in tobacco and that's great but…what is in the actual oils [e-liquids] and is that potentially doing harm? *C1-CS-EV*

2. Am I any better off? We won't know for 20 years." C2-NS-NV

3. "I would say ninety-five per cent of them use e-cigarettes, ninety percent or more..." *G2–NS-NV*"

4. "If you went to somebody's cell and it's a spontaneous job and somebody's vaping in the cell, how realistic is it for you to say…'You need to stop vaping! You need to stop that for an hour before I come into this cell'? It's impossible." *O3-NS-NV*

3b) Misuse of e-cigarettes to take illegal drugs

5. "The instances of the prisoners being under the influence of NPS, has increased quite dramatically over the past couple of months." *L3-NS-NV*

6. "The thing that prisoners have got is time to figure out how to take drugs…not every prisoner is like that obviously. It's only a certain amount that smoke drugs…but they adapt a different way of doing it and the thing is, they've got time to figure that out." *O3-NS-NV*

For peer review only - http://bmjopen.bmj.com/site/about/guidelines.xhtml

7. "The vape's just good [for NPS use] because it's enclosed… and also how do you police 500, 400 and odd people, in some cases the bigger jails, you've got a thousand odd prisoners. How do you police every single person with a vape?" *B1-NS-NV*

8. "A lot of staff become ill because they've entered somebody's cell to speak to them and by the time they've left they've started to feel…it's [second-hand exposure to NPS] taken effect. So I'm quite conscious if I go to a cell. If it's smoky [filled with e-cigarette vapour], I'm not so keen to go in there…it feels like it's a matter of time given how many of my colleagues have been affected by it…Headaches, high blood pressure, heartrate zooming up, becoming shaky and unsteady. Staff have been really, really poorly with it." *C1-CS-ExV*

9. "It's a completely different workplace now, and this is definitely something new for me, in the years I've worked here I've never known this before." *O4-ExS-ExV*

Misuse of e-cigarettes among PiC to take illegal drugs

Concerns about the risks associated with exposure to e-cigarette vapour were amplified because participants in all prisons viewed (rechargeable) e-cigarettes as providing a new and effective way for some PiC to take new psychoactive substances (NPS) (Table 3b-5), particularly since some PiC had a lot of unoccupied time that could be spent on (devising) illicit activity (Table 3b-6). Whilst some noted the removal of lighters as part of smokefree policy may have increased NPS use (replacing cannabis which PiC were less able to ignite), one reported that the number of PiC being placed on observations for drug use had increased over several years and so current NPS use might only reflect an underlying trend.

Residential staff gave accounts of how some PiC adapted e-cigarette devices to enable the ingestion of vapour from NPS-soaked paper and noted the unpredictable and adverse effects of this practice on PiC. Residential staff also noted that NPS use was easily concealed within an e-cigarette, and often odourless, adding to concerns about 'policing' NPS use (Table 3b-

For peer review only - http://bmjopen.bmj.com/site/about/guidelines.xhtml

7). They often spoke of feeling vulnerable at work and sometimes when driving home, due to the risks and effects of (unwitting) second-hand exposures to NPS, especially when entering cells. Accounts of harms to themselves or colleagues because of involuntary inhalation of NPS as a bystander were described (Table 3b-8) and a few suggested the effects of NPS use in prison had significantly changed their work environment (Table 3b-9).

Value attached to e-cigarettes in prisons

E-cigarettes were viewed as having become an integral part of prison life over a relatively short space of time, serving similar functions to those previously fulfilled by tobacco, including as a valued commodity/form of prison currency. As with other 'alternative' currencies or valued items in prisons, this could create problems for staff and the prison system (as well as for PiC themselves).

Firstly, staff reported having to manage problems, including tensions on the halls and challenging behaviour when PiC ran out of e-liquids and wanted to borrow e-cigarette products from others (Table 4a-1). Borrowing often came at a price; it was reportedly not uncommon for PiC to have to repay over the amount borrowed, leading to debt and in some cases bullying, in particular of more vulnerable PiC (Table 4a-2). One participant suggested that whilst issues of borrowing and debt (and bullying) were encountered in relation to other products, e-cigarette related debts could grow relatively quickly (Table 4a-3). Borrowing tobacco (before the smokefree policy was introduced) was seen as somewhat easier for PiC to manage, because it could be lent in small amounts, whereas borrowing e-cigarette products involved the loan of an e-liquid or device, both of higher monetary value.

Table 4: The value attached to e-cigarettes in prisons, and long term nicotine use and smoking cessation

**4a The value attached to e-cigarettes in prisons**

For peer review only - http://bmjopen.bmj.com/site/about/guidelines.xhtml

1. "…boys scratching the door, 'Oh boss, can you get us any vapes?' And then they're shouting to the other guys, 'Anyone got any?' and they were handed a capsule over with wee dribbles in it to keep them going." *C2-NV-NS*

2. "You have to try and nip that in the bud [vulnerable PiC getting into e-cigarette related debts], if you catch it, straight away." *G1-ExS-NV*

3. "There are, obviously, other things that get the guys in debt, but this seems to be the quick way...to borrow one [refill], pay back two, [and] not have the money [to buy your own] the following week." *G1-ExS-NV*

4. "We've already had an incident whereby one of the girls thought that she had caught something from the misuse of the vapes and that immediately stopped within the hall, for as far as we can try and control it." *B1-NS-NV*

5. "We had one [case] this morning actually. Someone was screaming and shouting because her vape has been nicked." *C1-CS-ExV*

4b Long-term nicotine use and maintained smoking cessation

6. "I think the smoking cessation works well, but I think the motivation for the prisoners to stop smoking disappears when they can have vapes. I think they were more motivated [to quit] just before the ban because they couldn't get any tobacco." *C3-NS-NV*

7. "They've purchased these vapes, bought them in jail, the first time they've taken them out with them [after release from prison] they've kept a hold of them because they're not got the financial money to go and buy cigarettes." *B1-NS-NV*

8. "Very seldom do I come across a prisoner who wants to stop smoking. Those who are being released keep telling me they're going over to the shop across the road to buy a pack of cigarettes when they get out. So they've got no intention of...the vape is just a short term thing for a lot of them." *G2-NS-NV*

4c Perceived potential solutions to the challenges raised by vaping in prisons

For peer review only - http://bmjopen.bmj.com/site/about/guidelines.xhtml

9. "because at the minute ….we're just giving it [e-cigarette products] out…there's nothing else other than 'vape, carry on vaping'." *G1-ExS-NV*

10. "Do the human rights of a prisoner outweigh the [human rights of the] guys who are working here?" *O3-NV-NS*

Secondly, the price of e-cigarette products was perceived to be a moderate or serious burden for some PiC, especially those on very low incomes. This sometimes led to the sharing of e-cigarette devices, and the exchange of part-used e-liquids (to enable use of different flavours at no additional cost) which raised concerns for staff about possible virus and disease transmission (Table 4a-4). (Note: interviews were conducted over a year before the first documented Covid-19 cases.)

There were several other ways that e-cigarettes created additional challenges for staff; for example, when PiC misplaced their device, the onus was on staff to break from their duties to locate it, and when e-cigarettes were reported as stolen (Table 4a-5). Several staff reported regular vaping in prohibited areas, and whilst some described this as unintentional, others reported instances where PiC had smuggled e-cigarettes outwith their cells, or had refused to go to appointments unless they were able to take their e-cigarettes with them. Some staff were willing to put PiC on report for repeated breaches, but others viewed the policing of e-cigarettes as too difficult because non-compliance was commonplace.

<u>Long-term tobacco and nicotine use</u>

Staff reported that prior to the introduction of e-cigarettes, some PiC had intended to become nicotine-free once smokefree rules were implemented. However, few PiC had reportedly achieved this; most previous smokers were vaping e-liquids containing nicotine long after tobacco had been removed from prisons (Table 4b-6). Staff were generally unsure about

For peer review only - http://bmjopen.bmj.com/site/about/guidelines.xhtml

what support was available in prisons for PiC who wished to quit vaping and noted that e-liquids were only available in higher strengths in prisons, which some staff viewed as a potential significant barrier to enabling PiC to reduce nicotine dependence. While staff noted that some individuals might continue to vape exclusively after leaving prison, for example for financial reasons (Table 4b-7), several relayed discussions with PiC who intended to smoke tobacco on release, and in some cases this was considered a *fait accompli* (Table 4b-8), reflecting, for example, people's intentions to resume co-use of cannabis and tobacco.

Perceived potential solutions to the challenges raised by vaping in prisons

Participants expressed varied views on solutions to the perceived challenges of vaping in prisons. These included only selling single use e-cigarettes in prisons to limit product misuse; and supporting PiC to manage and, if desired, reduce use of rechargeable e-cigarettes (Table 4c-9). A few participants suggested e-cigarettes should be completely prohibited in prisons, with one suggesting this would better balance the 'rights' of staff with those of PiC (Table 4c-10). However, staff acknowledged that greater vaping restrictions were likely to be unpopular with PiC, and very challenging to implement given the scale and significance of vaping in prisons among PiC.

DISCUSSION

This study provides novel insights into prison staff experiences of the challenges associated with allowing e-cigarettes to be sold and used among PiC in smokefree prisons, alongside further evidence of perceived individual and organisational benefits of e-cigarettes in prisons. Findings suggest that staff were concerned, to varying degrees, by perceived negative impacts of e-cigarettes on worker health and safety, job tasks, and work environment. Given that the roles of prison staff are highly demanding [15], it is perhaps unsurprising that participants were very alert to potential risks associated with this major organisational change while

For peer review only - http://bmjopen.bmj.com/site/about/guidelines.xhtml

generally expressing support, in national surveys, for having e-cigarettes in prisons for PiC.[4].

An area of concern to participants was the potential health impacts of (long-term) exposure to e-cigarette vapour at work. These concerns are particularly understandable given that rates of vaping are around 10 times higher among PiC than the Scottish general population [16, 17] and that prison staff need to enter rooms where PiC regularly vape, to provide urgent care or maintain security. Prisons appear unusual among UK public sector organisations in permitting use of e-cigarettes indoors; a recent study found that vaping was prohibited indoors in all NHS Trusts and almost all Higher Education Institutions.[18] Differences in vaping policies between prisons and other workplaces may be viewed particularly negatively by prison staff (at a symbolic and/or practical level) given that the UK prison workforce was exposed to SHS for over a decade longer than other UK workforces because prisons were partially exempt from Scotland's 2006 national smokefree laws. Concerns among prison staff in Scotland about the potential health effects of e-cigarettes on bystanders (and users) may also reflect broader anxieties about the safety of e-cigarettes; similar concerns have been expressed by other occupational groups e.g. healthcare staff.[19] Amongst the general population, a US study found that participants generally perceived e-cigarette vapour exposures to be '*moderately harmful'* to health; almost half believed that vaping should be prohibited in indoor public places such as restaurants.[20]

The use of e-cigarettes for illicit drug use is understandably of significant concern to prison staff given the adverse impacts that this can have on those living and working in prison and operational stability.[21] There is a growing body of evidence on the challenges and harms posed by NPS use in prisons.[22-24] Understandings of the general risks posed by e-cigarettes for illicit drug taking, including NPS, are developing: a 2018 systematic review [25] suggested several potential areas of risk. These include: that e-cigarettes might be

For peer review only - http://bmjopen.bmj.com/site/about/guidelines.xhtml

viewed as a '*safer and innocuous method to experiment and try drugs*'; levels of illicit drug use might increase due to the ease with which e-cigarettes can be used for drug delivery; the emergence of new patterns of drug use; and illicit drug use may be easier to conceal, leading to increased problems with detection by authorities and greater risks of '*unintended or malicious'* exposures to bystanders (p107). It will be important to monitor these risks, including in prison settings. These findings have implications for the choice and design of rechargeable e-cigarette devices, including those used in prisons and other secure settings such as in-patient mental health services.

Prison staff also discussed challenges related to the value attached to e-cigarettes in prisons. These appeared similar to those previously caused by tobacco prior to smokefree prison policy and are to be expected, given prison rules on purchasing items from the prison shop and the informal economy in prisons.[26] (Analysis of canteen (prison shop) spend data on the relative costs of smoking and vaping in prisons is underway and will be reported separately.) Nonetheless, some staff might view such issues as particularly troublesome at a time when the workforce is facing wider challenges including increasing numbers of PiC, staff sickness absence rates and increased working hours.[27]

Finally, staff reported potential limitations or challenges of e-cigarettes for reducing tobacco harms in PiC long-term. Discomfort with continued use of nicotine among PiC may stem from staff knowledge that some individuals have a strong desire to become 'free' of substance dependence, as well as from (mis)understandings of health risks of nicotine [28] or perceptions that someone who vapes has not truly stopped 'smoking'.[29] Staff members' acknowledgement of risks that many PiC might resume smoking soon after leaving smokefree prisons, despite having exclusively vaped for a period, reflects the substantial challenges that people often face in transitioning from prison to the outside world.[30]

For peer review only - http://bmjopen.bmj.com/site/about/guidelines.xhtml

Findings reported here will be of interest to jurisdictions planning on making their prison systems smokefree and those considering use of e-cigarettes for harm reduction. They highlight the need for any policies on e-cigarette use in prisons to balance the needs of those PiC who wish to use e-cigarettes (in lieu of tobacco) against calls for precautionary measures to limit (or eliminate) e-cigarette vapour exposures because of a lack of long-term evidence on e-cigarette safety. Based on current evidence regarding their effects on air quality and health [31], restricting indoor vaping in prisons to private rooms (cells) would appear proportionate, although there is a need for ongoing monitoring of the evidence and clear communication for the rationale for policies to prison staff. Like smoking previously, where allowed, vaping in prisons is likely to become normative and culturally embedded over time, potentially presenting future challenges for a prison service should the need to introduce tighter restrictions on e-cigarette use arise. Other challenges associated with the use of e-cigarettes among PiC might be reduced through providing guidance and support on effective use and on how to limit or quit use if desired (e.g., see practitioner guidance developed in Scotland, subsequent to these staff interviews, to support PiC to reduce or cease use of e-cigarettes [32]) and smoking relapse prevention interventions spanning the pre-post release period. Broader responses are also required to tackle illicit drug use among PiC, including measures to reduce environmental drivers of prison drug use.[33]

We believe the use of one-to-one interviews in this study is a strength because they enable in-depth exploration of people's perspectives and of confidential or sensitive issues [34]. Findings from this study complement and extend our previous work exploring staff perspectives on e-cigarettes using data gathered via focus groups before announcement of the smokefree policy,[8] and again post implementation (Brown 2021, submitted). A key study limitation is that certain participant groups (e.g. females, current vapers) may not be adequately represented in the sample, limiting our ability to explore potential between-group

For peer review only - http://bmjopen.bmj.com/site/about/guidelines.xhtml

differences in perspectives. However, the inclusion of staff from a diverse set of prisons provides confidence that the breadth of challenges associated with e-cigarettes in prisons are likely to be reflected well in the data. It is important to note that data were collected prior to the introduction of guidance for practitioners on how to support PiC to reduce or stop using e-cigarettes and before the COVID-19 pandemic, both of which may have important implications for vaping in prisons and are priorities for future studies.

In conclusion, this detailed analysis, focused on the *challenges* associated with vaping in prisons from the perspective of prison staff, identified five main issues: staff concerns about exposures to e-cigarette vapour at work; use of e-cigarettes for illicit drug taking among PiC; the value attached to, and trading of, e-cigarette products in prisons; and implications for long-term nicotine and tobacco use. Addressing these challenges is likely to require a combination of measures specific to e-cigarettes and broader measures to address (illicit) drug use and promote overall health.

Acknowledgements

We are grateful to the prison staff who took part in the interviews, staff within the Scottish Prison Service who assisted with the study and facilitated access, and NHS staff who have supported our research on smoke-free prisons.

Contributors

KH, AB, HS and LB developed the study, with input from RO, DE, RP and the SPS Research Advisory Group. AB and KH liaised with the prison service and reported back on ongoing findings. AB prepared the ethics applications. RO, AB and DE conducted the qualitative interviews; all are highly experienced qualitative researchers. AB, RO and DM conducted analysis using the Framework approach. RO, AB and KH drafted the manuscript and all

For peer review only - http://bmjopen.bmj.com/site/about/guidelines.xhtml

revisions. All other authors read a subsample of the transcripts and contributed to interpretation of the data and review of the final manuscript.

Funding

The study was supported by Cancer Research UK (C45874/A27016). ED and HS were supported by the UK Medical Research Council MC_UU_12017/12 and Scottish Chief Scientist Office SPHSU12.

Declarations of interest

None to declare

Ethical approval

Ethical approval was granted by the SPS Research Access and Ethics Committee (approval confirmed 23.8.18; no ref number) and by the University of Stirling General University Ethics Panel (ref: GUEP 497).

Data availability statement

No additional data available.

For peer review only

References

[1] Brown A, O'Donnell R, Eadie D*, et al.* Initial Views and Experiences of Vaping in Prisons: A Qualitative Study With People in Custody Preparing for the Imminent Implementation of Scotland's Prison Smokefree Policy. *Nicotine & Tobacco Research* 2020.

[2] Woodall J, Tattersfield A. Perspectives on implementing smoke-free prison policies in England and Wales. *Health Promot Int* 2018;**33**(6):1066-1073.

[3] Kennedy SM, Davis SP, Thorne SL. Smoke-free policies in U.S. Prisons and jails: A review of the literature. *Nicotine Tob Res* 2015;**17**(6):629-635.

[4] Sweeting H, Demou E, Brown A*, et al.* Prisoners and prison staff express increased support for prison smoking bans following implementation across Scotland: results from the Tobacco In Prisons study. *Tobacco Control* 2020:tobaccocontrol-2020-055683.

[5] Beard E, West R, Michie S*, et al.* Association between electronic cigarette use and changes in quit attempts, success of quit attempts, use of smoking cessation pharmacotherapy, and use of stop smoking services in England: time series analysis of population trends. *BMJ* 2016;**354**:i4645.

[6] Hartmann-Boyce J, McRobbie H, Lindson N*, et al.* Electronic cigarettes for smoking cessation. . *Cochrane Database of Systematic Reviews* 2020;**Issue 10**.

[7] National Academies of Sciences Engineering and Medicine. Public Health Consequences of E-Cigarettes. Washington (DC): National Academies Press (US). 2018.

[8] Brown A, Sweeting H, Semple S*, et al.* Views of prison staff in Scotland on the potential benefits and risks of e-cigarettes in smoke-free prisons: a qualitative focus group study. *BMJ Open* 2019;**9**(6):e027799.

[9] Brown A, O'Donnell R, Eadie D*, et al.* E-cigarette use in prisons with recently established smokefree policies: a qualitative interview study with people in custody in Scotland. *Nicotine & Tobacco Research* 2020.

For peer review only - http://bmjopen.bmj.com/site/about/guidelines.xhtml

[10] Curry L, Lee YO, Rogers T. E-cigarettes made especially for inmates. *Tobacco control* 2014;**23**(e2):e87-e88.

[11] Young-Wolff KC, Karan LD, Prochaska JJ. Electronic cigarettes in jails: a panacea or public health problem? *JAMA Psychiatry* 2015;**72**(2):103-104.

[12] Brown A, Sweeting H, Logan G*, et al.* Prison Staff and Prisoner Views on a Prison Smoking Ban: Evidence From the Tobacco in Prisons Study. *Nicotine & Tobacco Research* 2018;**21**(8):1027-1035.

[13] Spencer L RJ, O'Connor W, Morrell G, Ormston R. . Analysis in Practice. In: Ritchie J LJ, McNaughton Nicholls C, Ormston R, ed. *Qualitative Research Practice* Sage 2014:295-343.

[14] Gale NK, Shapiro J, McLeod HS*, et al.* Patients-people-place: developing a framework for researching organizational culture during health service redesign and change. *Implement Sci* 2014;**9**:106.

[15] Kinman G, Clements AJ, Hart J. Job demands, resources and mental health in UK prison officers. *Occupational Medicine* 2017;**67**(6):456-460.

[16] McLean J, Dean L. Scottish Health Survey 2018: main report - revised 2020. 2020.

[17] Scottish Prison Service. Prisoner Survery 2019. Scottish Prison Service 2019.

[18] Blackwell AKM, Kosīte D, Marteau TM*, et al.* Policies for Tobacco and E-Cigarette Use: A Survey of All Higher Education Institutions and NHS Trusts in England. *Nicotine & Tobacco Research* 2020;**22**(7):1235-1238.

[19] Stepney M, Aveyard P, Begh R. GPs’ and nurses’ perceptions of electronic cigarettes in England: a qualitative interview study. *British Journal of General Practice* 2019;**69**(678):e8.

For peer review only - http://bmjopen.bmj.com/site/about/guidelines.xhtml

[20] Mello S, Bigman CA, Sanders-Jackson A*, et al.* Perceived Harm of Secondhand Electronic Cigarette Vapors and Policy Support to Restrict Public Vaping: Results From a National Survey of US Adults. *Nicotine & Tobacco Research* 2016;**18**(5):686-693.

[21] Kolind T, Duke K. Drugs in prisons: Exploring use, control, treatment and policy. *Drugs: Education, Prevention and Policy* 2016;**23**(2):89-92.

[22] Ralphs R, Williams L, Askew R*, et al.* Adding Spice to the Porridge: The development of a synthetic cannabinoid market in an English prison. *International Journal of Drug Policy* 2017;**40**:57-69.

[23] Corazza O, Coloccini S, Marrinan S*, et al.* Novel Psychoactive Substances in Custodial Settings: A Mixed Method Investigation on the Experiences of People in Prison and Professionals Working With Them. *Frontiers in Psychiatry* 2020;**11**(460).

[24] Gooch K, Treadwell J. Prisoner Society in an Era of Psychoactive Substances, Organized Crime, New Drug Markets and Austerity. *The British Journal of Criminology* 2020;**60**(5):1260-1281.

[25] Breitbarth AK, Morgan J, Jones AL. E-cigarettes—An unintended illicit drug delivery system. *Drug and Alcohol Dependence* 2018;**192**:98-111.

[26] Walker DF. The informal economy in prison. *Criminal Justice Matters* 2015;**99**(1):18-19.

[27] Auditor General. The 2018/19 audit of the Scottish Prison Service. 2020.

[28] O'Brien EK, Nguyen AB, Persoskie A*, et al.* U.S. adults' addiction and harm beliefs about nicotine and low nicotine cigarettes. *Prev Med* 2017;**96**:94-100.

[29] Hartwell G, Egan M, Petticrew M. Understanding decisions to use e-cigarettes or behavioural support to quit tobacco: a qualitative study of current and ex-smokers and stop smoking service staff. *Addiction* 2020;**115**(3):518-526.

For peer review only - http://bmjopen.bmj.com/site/about/guidelines.xhtml

[30] Puljevic C, Segan CJ. Systematic Review of Factors Influencing Smoking Following Release From Smoke-Free Prisons. *Nicotine Tob Res* 2019;**21**(8):1011-1020.

[31] Li L, Lin Y, Xia T, *et al.* Effects of Electronic Cigarettes on Indoor Air Quality and Health. *Annu Rev Public Health* 2020;**41**:363-380.

[32] NHS Health Scotland. Supporting individuals to cut down and quit vaping: Guidance for NHS Quit Your Way advisers based in prisons. 2020.

[33] Mjåland K. Exploring prison drug use in the context of prison-based drug rehabilitation. *Drugs: Education, Prevention and Policy* 2016;**23**(2):154-162.

[34] Guest G, Namey, E. & Mitchell, M. . In-depth interviews. *Collecting Qualitative Data*: SAGE Publications, Ltd. 2013:113-171.

For peer review only

For peer review only - http://bmjopen.bmj.com/site/about/guidelines.xhtml

COREQ (COnsolidated criteria for REporting Qualitative research) Checklist

A checklist of items that should be included in reports of qualitative research. You must report the page number in your manuscript where you consider each of the items listed in this checklist. If you have not included this information, either revise your manuscript accordingly before submitting or note N/A.

Topic	Item No.	Guide Questions/Description	Reported on Page No.
**Domain 1: Research team and reflexivity**			
Personal characteristics			
Interviewer/facilitator	1	Which author/s conducted the interview or focus group?	
Credentials	2	What were the researcher's credentials? E.g. PhD, MD	
Occupation	3	What was their occupation at the time of the study?	
Gender	4	Was the researcher male or female?	
Experience and training	5	What experience or training did the researcher have?	
Relationship with participants			
Relationship established	6	Was a relationship established prior to study commencement?	
Participant knowledge of the interviewer	7	What did the participants know about the researcher? e.g. personal goals, reasons for doing the research	
Interviewer characteristics	8	What characteristics were reported about the inter viewer/facilitator? e.g. Bias, assumptions, reasons and interests in the research topic	
**Domain 2: Study design**			
Theoretical framework			
Methodological orientation and Theory	9	What methodological orientation was stated to underpin the study? e.g. grounded theory, discourse analysis, ethnography, phenomenology, content analysis	
Participant selection			
Sampling	10	How were participants selected? e.g. purposive, convenience, consecutive, snowball	
Method of approach	11	How were participants approached? e.g. face-to-face, telephone, mail, email	
Sample size	12	How many participants were in the study?	
Non-participation	13	How many people refused to participate or dropped out? Reasons?	
Setting			
Setting of data collection	14	Where was the data collected? e.g. home, clinic, workplace	
Presence of non-participants	15	Was anyone else present besides the participants and researchers?	
Description of sample	16	What are the important characteristics of the sample? e.g. demographic data, date	
Data collection			
Interview guide	17	Were questions, prompts, guides provided by the authors? Was it pilot tested?	
Repeat interviews	18	Were repeat inter views carried out? If yes, how many?	
Audio/visual recording	19	Did the research use audio or visual recording to collect the data?	
Field notes	20	Were field notes made during and/or after the inter view or focus group?	
Duration	21	What was the duration of the inter views or focus group?	
Data saturation	22	Was data saturation discussed?	
Transcripts returned	23	Were transcripts returned to participants for comment and/or	

For peer review only

Topic	Item No.	Guide Questions/Description	Reported on Page No.
		correction?	
**Domain 3: analysis and findings**			
Data analysis			
Number of data coders	24	How many data coders coded the data?	
Description of the coding tree	25	Did authors provide a description of the coding tree?	
Derivation of themes	26	Were themes identified in advance or derived from the data?	
Software	27	What software, if applicable, was used to manage the data?	
Participant checking	28	Did participants provide feedback on the findings?	
Reporting			
Quotations presented	29	Were participant quotations presented to illustrate the themes/findings? Was each quotation identified? e.g. participant number	
Data and findings consistent	30	Was there consistency between the data presented and the findings?	
Clarity of major themes	31	Were major themes clearly presented in the findings?	
Clarity of minor themes	32	Is there a description of diverse cases or discussion of minor themes?	

Developed from: Tong A, Sainsbury P, Craig J. Consolidated criteria for reporting qualitative research (COREQ): a 32-item checklist for interviews and focus groups. *International Journal for Quality in Health Care*. 2007. Volume 19, Number 6: pp. 349 – 357

Once you have completed this checklist, please save a copy and upload it as part of your submission. DO NOT include this checklist as part of the main manuscript document. It must be uploaded as a separate file.

For peer review only

BMJ Open

The challenges associated with e-cigarette use by people in custody in Scottish prisons: a qualitative interview study with prison staff

Journal:	*BMJ Open*
Manuscript ID	bmjopen-2021-051009.R1
Article Type:	Original research
Date Submitted by the Author:	04-Oct-2021
Complete List of Authors:	O'Donnell, Rachel; University of Stirling, Institute of Social Marketing and Health<br>Brown, Ashley; University of Stirling Institute for Social Marketing<br>Eadie, Douglas; Institute for Social Marketing, School of Health Sciences<br>Mitchell, Danielle; Institute for Social Marketing and Health, University of Stirling, Faculty of Health Sciences and Sport<br>Bauld, Linda; University of Edinburgh, Usher Institute and UK Centre for Tobacco and Alcohol Studies, College of Medicine and Veterinary Medicine<br>Demou, Evangelia; University of Glasgow, MRC/CSO Social & Public Health Sciences Unit<br>Purves, Richard; University of Stirling, Institute for Social Marketing and Health<br>Sweeting , Helen ; University of Glasgow, MRC/CSO Social & Public Health Sciences Unit<br>Hunt, Kate; University of Stirling Institute for Social Marketing and Health, Institute for Social Marketing and Health
**Primary Subject Heading**:	Public health
Secondary Subject Heading:	Smoking and tobacco
Keywords:	PUBLIC HEALTH, QUALITATIVE RESEARCH, OCCUPATIONAL & INDUSTRIAL MEDICINE

SCHOLARONE™
Manuscripts

I, the Submitting Author has the right to grant and does grant on behalf of all authors of the Work (as defined in the below author licence), an exclusive licence and/or a non-exclusive licence for contributions from authors who are: i) UK Crown employees; ii) where BMJ has agreed a CC-BY licence shall apply, and/or iii) in accordance with the terms applicable for US Federal Government officers or employees acting as part of their official duties; on a worldwide, perpetual, irrevocable, royalty-free basis to BMJ Publishing Group Ltd ("BMJ") its licensees and where the relevant Journal is co-owned by BMJ to the co-owners of the Journal, to publish the Work in this journal and any other BMJ products and to exploit all rights, as set out in our licence.

The Submitting Author accepts and understands that any supply made under these terms is made by BMJ to the Submitting Author unless you are acting as an employee on behalf of your employer or a postgraduate student of an affiliated institution which is paying any applicable article publishing charge ("APC") for Open Access articles. Where the Submitting Author wishes to make the Work available on an Open Access basis (and intends to pay the relevant APC), the terms of reuse of such Open Access shall be governed by a Creative Commons licence – details of these licences and which Creative Commons licence will apply to this Work are set out in our licence referred to above.

Other than as permitted in any relevant BMJ Author's Self Archiving Policies, I confirm this Work has not been accepted for publication elsewhere, is not being considered for publication elsewhere and does not duplicate material already published. I confirm all authors consent to publication of this Work and authorise the granting of this licence.

For peer review only

The challenges associated with e-cigarette use by people in custody in Scottish prisons: a qualitative interview study with prison staff

Rachel O'Donnell[1*], Ashley Brown[1*], Douglas Eadie[1], Danielle Mitchell[1], Linda Bauld[2], Evangelia Demou[3], Richard Purves[1], Helen Sweeting[3] and Kate Hunt[1]

1. Institute for Social Marketing and Health, Faculty of Health Sciences and Sport, University of Stirling, Pathfoot Building, Stirling, FK9 4LA.

2. Usher Institute and SPECTRUM Consortium, College of Medicine and Veterinary Medicine, University of Edinburgh.

3. MRC/CSO Social and Public Health Sciences Unit, Berkeley Square, 99 Berkeley Street, Glasgow, G3 7HR.

* RO and AB are joint first authors and contributed equally to this work.

Corresponding author: Rachel O'Donnell r.c.odonnell@stir.ac.uk

Word Count: 3815/4000 words

Keywords: e-cigarettes, vaping, prisoner health, staff, workplace, qualitative

For peer review only - http://bmjopen.bmj.com/site/about/guidelines.xhtml

ABSTRACT

Objectives: Little is known about the perspectives of staff working in prisons where e-cigarettes are permitted. Scotland now permits people in custody (PiC), but not staff/visitors to use e-cigarettes, following implementation of smokefree prisons policy in 2018. Previous studies, conducted before and immediately after the introduction of e-cigarettes in Scottish prisons, have evidenced stakeholder support for their use by PiC. This study focuses on key challenges associated with e-cigarette use in prisons, using data collected from prison staff once e-cigarettes had been allowed in a smoke-free environment for 6-9 months.

Setting: Five prisons in Scotland.

Participants: Sixteen qualitative interviews were conducted with prison staff from five prisons varying by population (sex, age and sentence length). Data were managed and analysed using the Framework approach.

Results: Whilst these staff confirmed strong support for the smokefree prison policy and reported some benefits of replacing tobacco with e-cigarettes, they also spoke of the challenges e-cigarettes pose. These included: workplace e-cigarette vapour exposures; perceptions that e-cigarettes provide a new, effective way for some PiC to take illegal drugs, particularly new psychoactive substances; organisational challenges relating to the value attached to e-cigarettes in prisons; and implications for long-term nicotine use and tobacco cessation. Staff anticipated difficulties in tightening restrictions on e-cigarette use by PiC given its scale and significance among this population.

Conclusions: Maximizing the benefits of e-cigarette use by PiC is likely to require multiple measures to support effective and safe use and e-cigarette reduction/cessation where desired. This includes monitoring any misuse of e-cigarettes, and provision of guidance and support on appropriate e-cigarette use and how to limit or quit use if desired. Findings are relevant to jurisdictions considering or planning changes in prison smoking or vaping policies.

For peer review only - http://bmjopen.bmj.com/site/about/guidelines.xhtml

Strengths and limitations of the study

- This qualitative study provides in-depth insights into prison staff experiences of the challenges associated with allowing e-cigarettes to be sold and used among people in custody in prisons with established smokefree policies.
- This study broadens understanding of risks and challenges of use of e-cigarettes in this distinct setting and corresponding measures that might minimise harms and maximise benefits.
- The study findings highlight the need for policies on e-cigarette use in prisons to balance the needs of people in custody who wish to use e-cigarettes against concerns about exposures to e-cigarette vapour at work; use of e-cigarettes for illicit drug taking among people in custody; the value and trading of e-cigarette products in prisons; and implications for long-term nicotine and tobacco use.
- Whilst some staff groups (e.g. female members of staff, current vapers) may not be adequately represented in the sample, the inclusion of staff from a diverse set of prisons provides confidence that the breadth of challenges associated with e-cigarettes in prisons are likely to be reflected.

Funding statement: This work was supported by Cancer Research UK (C45874/A27016). ED and HS were also supported by the UK Medical Research Council MC_UU_12017/12 and Scottish Chief Scientist Office SPHSU12.

Competing interests statement: None to declare

For peer review only - http://bmjopen.bmj.com/site/about/guidelines.xhtml

INTRODUCTION

In anticipation of the November 2018 implementation of the country's comprehensive (indoor/outdoor) smokefree prison policy, legislative changes occurred which enabled people in custody (PiC) in Scotland to purchase and use rechargeable e-cigarettes in rooms (cells) and outdoor spaces from September 2018. Following their introduction, the places where e-cigarette use is permitted mirrors the places where PiC were allowed to smoke tobacco prior to implementation of the smokefree policy. Tobacco stopped being sold in prisons via the prison shop ('canteen') ~two weeks before the smokefree policy was introduced. Prison staff and visitors remained prohibited from smoking, or using e-cigarettes (hereafter vaping) on prison property. Information on e-cigarette products sold via the canteen in Scottish prisons and prison smoking cessation services is available elsewhere.[1, 2] The introduction of e-cigarettes in prisons in Scotland (and in other jurisdictions such as England and Wales, and the US [3, 4]) has potentially important implications for the health and safety of PiC and prison staff, and the operation of prison systems. There is very limited evidence on e-cigarette use in prisons, with no studies reporting on the perspectives of staff working in prisons where vaping is permitted among PiC, thus limiting understandings of its perceived benefits and risks in this distinct setting.

Current research evidence on e-cigarette use in prisons comes from two studies of tobacco and vaping in Scottish prisons (the Tobacco in Prisons study [TIPs] and the E-cigarettes in Prisons study). Evidence from TIPs, and responses to a public consultation on smokefree prisons (by the Scottish Prison Service), showed that e-cigarettes received broad support from stakeholders in anticipation of the introduction of smokefree prison policy. For example, TIPs surveys in May-June 2018 (after the smokefree policy announcement but before it was known e-cigarettes would be made available to PiC) and May-June 2019 (6-9 months after use of (rechargeable) e-cigarettes was first allowed and smokefree policy was implemented in

For peer review only

Scottish prisons) both showed the majority of prison staff (~75%) and almost all PiC (~90%) agreed that ‘e-cigarettes should be available to help prisoners to stop smoking/manage without tobacco’.[5]

Complementary qualitative research suggests this broad support for e-cigarettes in Scottish prisons has been substantially influenced by perceptions that they are beneficial in helping PiC and staff to cope with the major operational change represented by the removal of tobacco.[6] Favourable perceptions of e-cigarettes in Scottish prisons also reflect broader evidence on their appeal and effectiveness for some current and ex-smokers (e.g. those who feel unwilling or unable to quit nicotine)[7, 8] and evidence on reduced harms to users and bystanders from vaping relative to smoking.[9]

However, important issues and challenges in relation to permitting PiC to vape were also voiced by prison staff in anticipation of their introduction [8] (and some PiC in anticipation of and again 6-10 months after their implementation [1, 10, 11], as well as by commentators in other jurisdictions.[12, 13] These concerns relate to e-cigarette safety, implications for long-term tobacco and nicotine cessation (e.g. uncertainties about whether e-cigarette use in prison increases or decreases the likelihood of return to smoking on release), and risks associated with vaping related to unique features of prison environments. These include: rules around earnings and purchases that mean PiC are not always able to (formally) buy (enough of) the items they want or ‘need’; operation of illicit prison economies in which any sought-after item can be traded or ‘bought’ using alternative currencies; and well established practices of PiC repurposing items, including for illicit purposes such as drug taking.[1] The benefits of e-cigarettes in prisons are well covered in our previous publications (including focus groups with staff before rules were changed to allow PiC to vape;[11, 14] interviews with PiC immediately after e-cigarettes were introduced [1] and again once new rules on e-cigarettes and smoking had bedded in;[10] and repeat cross sectional surveys of staff and

For peer review only

PiC[5]). This paper therefore focuses on the *challenges* of e-cigarettes in prisons identified by prison staff, based on novel interview data collected after vaping in prisons had become well established (8-11 months after sales of rechargeable e-cigarettes first began in Scottish prisons and 6-9 months after smokefree rules were implemented). Together with our previous studies, the current findings will be relevant for prison systems (and institutions such as mental health inpatient services) seeking to implement, and maximise the benefits of, smokefree policies.

METHODS

Patient and Public Involvement

The study was designed to ensure that the views of prison staff could be heard at different stages of the process of implementing smoke-free prisons in Scotland. A Research Advisory Group which included representation from prison management and union members provided feedback on the overall design of the study, study materials, and early findings to inform data collection and interpretation. Staff within the prisons were involved in the dissemination of information about the study and recruitment to the study. Personnel involved in implementing and managing smoke-free policies were also involved in discussion of findings to establish recommendations for future policy-makers and implementers.

Sample and recruitment

Data were collected between May-August 2019 from 16 Scottish Prison Service staff working in five (of Scotland's 15) prisons which varied by prison population (sex, age and sentence length) and capacity. Participants were recruited through designated staff contacts who were asked to invite staff from a range of work roles (e.g. residential; admissions; health and safety; prison management), to enable the exploration of diverse views. Our contacts

For peer review only - http://bmjopen.bmj.com/site/about/guidelines.xhtml

scheduled one-to-one meetings between researchers and staff who expressed interest in participation. Sample characteristics are detailed in Table 1.

Table 1: Participant Characteristics

**Work role**		**Number of years of service**	
Residential officer	8	1-10	2
Non-residential officer	6	11-20	6
Managerial	1	21-30	5
Missing	1	31-40	2
		Missing	1
**Sex**		**Age (years)**	
Male	13	18-30	2
Female	3	31-40	2
		41-50	3
		51-60	8
		Missing	1
**Smoking status**		**Vaping status**	
Non smoker (NS)	9	Non vaper (NV)	11
Ex smoker (ExS)	4	Ex vaper (ExV)	3
Current smoker (CS)	2	Current vaper (CV)	1
Missing	1	Missing	1

Data collection

Interviews (averaging 35 minutes) were conducted in a room which allowed the participant to speak freely. After providing information about the study and answering any questions,

For peer review only - http://bmjopen.bmj.com/site/about/guidelines.xhtml

written or audio-recorded verbal consent was obtained from each participant by one of three interviewers (RO, AB, DE) before s/he commenced the interview. The topic guide covered: participant background, smoking and vaping history; views and experiences related to vaping in the prison context; and views on the positive and negative consequences of vaping by PiC for prison staff, the prison regime and PiC themselves. Question wording and order were varied as appropriate and participants were invited to raise any additional points they considered important.

Data analysis

Fifteen of the 16 interviews were audio-recorded and transcribed by a professional transcription agency; detailed notes were made in lieu of audio-recording of one interview on participant request. All interview content was de-identified, then analysed (by RO, AB, DM) using the Framework approach.[15] This approach is well suited to the analysis of in-depth interview data, as summarisation of data into a framework grid within the NVivo program facilitates analysis across cases without losing the wider context of each participant's account.[16] A thematic framework was developed (by AB, RO) to guide data management and analysis, based on deductive (considering the topic guide, relevant literature) and inductive (reading transcripts) techniques. In preparation for detailed analysis, data summaries were written in relevant cells of the framework grid (RO, DM, AB), incorporating hyperlinks to transcripts to facilitate data retrieval. RO reviewed all summaries to check data interpretation and consistency of approach. Data summaries were then used to identify high level themes (AB, RO) before further in-depth analysis was conducted (RO), focusing on data about challenges posed by e-cigarettes in prisons. RO and AB finalised themes based on re-examining data and reflexive team discussions. Themes are presented below alongside illustrative quotes in Tables 2, 3 and 4; each quote indicates the prison worked in and the participant's interview ID number, and smoking and vaping status.

For peer review only - http://bmjopen.bmj.com/site/about/guidelines.xhtml

RESULTS

Staff perceptions of the smoke free prison policy and e-cigarette availability for PiC

Prior to reporting staff perspectives of the *challenges* of e-cigarettes in prisons, we briefly summarise, for context, their opinions on smokefree policy and beliefs about the benefits of e-cigarettes in prisons. Staff interviewed for this study voiced strong overall support for the smokefree prison policy and reported positive impacts of removing tobacco from prisons, including benefits for their own health and comfort while at work (Table 2-1) (see also [1, 11]). E-cigarettes were generally believed to have supported smokefree prison policy implementation (Table 2-2) and benefits for reducing tobacco-related harms among PiC and staff were sometimes discussed. However, whilst most participants acknowledged some benefits associated with the introduction of e-cigarettes in prisons, several drawbacks were also discussed, leading some to reflect on whether smokefree prisons could have been achieved without allowing PiC to vape (Table 2, 3-4).

Table 2: Staff perceptions of the smoke-free prison policy and e-cigarette availability

1.	"It's night and day in that the smell of tobacco's not there. You're not breathing it in. …it's not in your clothes. You're not bringing it home to your family. That to me is a big thing." *L4-NS-NV*
2.	"The fact that the e-cigarettes gave an alternative to the cigarettes actually made the transition of the jail changing over to no smoking a lot easier…it was a massive help to us." *B1-NS-NV*
3	"…making jails smoke free, we could just say, right that's it [no nicotine substitute], and we would have had bother [from PiC]. But would we have got through that bother, and then not had the smokers [vapers]?" *C2-NS-NV*

For peer review only - http://bmjopen.bmj.com/site/about/guidelines.xhtml

4	"The vapes made the process from smoking tobacco to there not being tobacco, it made that an easy process because we gave the prisoners a new option, while we kind of said to ourselves…'Why not just take the hit [i.e. without introducing e-cigarettes] and see how it goes with no tobacco?' Would that not have been better? Especially because I don't think there's enough research into the e-cigarettes and the vaping as well. We don't know exactly what kind of consequences there are." *O3-NS-NV*

Staff exposures to e-cigarette vapour

Several participants expressed doubts about the safety of vaping for themselves as bystanders (Table3a-1), in many cases referring to the absence of long-term studies on exposure to e-cigarette vapour (Table 3a-2). Concerns about exposure to e-cigarette vapour whilst at work were discussed in the context of very widespread vaping among PiC (Table 3a-3). In this context, some staff reported practical challenges in avoiding exposures to e-cigarette vapour when carrying out tasks such as cell searches (Table 3a-4). One participant wondered whether the persistent cough she had started to experience was linked to workplace exposure to e-cigarette vapour.

Table 3: Staff exposures to e-cigarette vapour, and misuse of e-cigarettes among PiC to take illegal drugs

**3a) Staff exposures to e-cigarette vapour**
1. "I wonder what the harm is of passive smoking with the vapes. I know we've taken away a lot of chemicals and tar and things that were in tobacco and that's great but…what is in the actual oils [e-liquids] and is that potentially doing harm? *C1-CS-EV*
2. Am I any better off? We won't know for 20 years." C2-NS-NV
3. "I would say ninety-five per cent of them use e-cigarettes, ninety percent or more..." *G2–NS-NV*"

For peer review only - http://bmjopen.bmj.com/site/about/guidelines.xhtml

4. “If you went to somebody’s cell and it’s a spontaneous job and somebody’s vaping in the cell, how realistic is it for you to say…‘You need to stop vaping! You need to stop that for an hour before I come into this cell’? It’s impossible.” *O3-NS-NV*

3b) Misuse of e-cigarettes to take illegal drugs

1. “There has been a lot of instances of vapes being broken, or improvised, shall we say …prisoners now know they can put paper in. It’s obviously soaked in NPS (novel psychoactive substances)…and that’s now their hit.” *L3-NS-NV*

2. “The thing that prisoners have got is time to figure out how to take drugs…not every prisoner is like that obviously. It’s only a certain amount that smoke drugs…but they adapt a different way of doing it and the thing is, they’ve got time to figure that out.” *O3-NS-NV*

3. “The vape’s just good [for NPS use] because it’s enclosed… and also how do you police 500, 400 and odd people, in some cases the bigger jails, you’ve got a thousand odd prisoners. How do you police every single person with a vape?” *B1-NS-NV*

4. “A lot of staff become ill because they’ve entered somebody’s cell to speak to them and by the time they’ve left they’ve started to feel…it’s [second-hand exposure to NPS] taken effect. So I’m quite conscious if I go to a cell. If it’s smoky [filled with e-cigarette vapour], I’m not so keen to go in there…it feels like it’s a matter of time given how many of my colleagues have been affected by it…Headaches, high blood pressure, heartrate zooming up, becoming shaky and unsteady. Staff have been really, really poorly with it.” *C1-CS-ExV*

5. “It’s a completely different workplace now, and this is definitely something new for me, in the years I’ve worked here I’ve never known this before.” *O4-ExS-ExV*

<u>Misuse of e-cigarettes among PiC to take illegal drugs</u>

For peer review only - http://bmjopen.bmj.com/site/about/guidelines.xhtml

Concerns about the risks associated with exposure to e-cigarette vapour were amplified because participants in all prisons viewed (rechargeable) e-cigarettes as providing a new and effective way for some PiC to take NPS (Table 3b-1), particularly since some PiC had a lot of unoccupied time that could be spent on (devising) illicit activity (Table 3b-2). Whilst some noted the removal of lighters as part of smokefree policy may have contributed to increased NPS use (replacing cannabis which PiC were less able to ignite), one reported that the number of PiC being placed on observations for drug use had increased over several years and so current NPS use might only reflect an underlying trend.

Residential staff gave accounts of how some PiC adapted e-cigarette devices to enable the ingestion of vapour from NPS-soaked paper and noted the unpredictable and adverse effects of this practice on PiC. Residential staff also noted that NPS use was easily concealed within an e-cigarette, and often odourless, adding to concerns about 'policing' NPS use (Table 3b-3). They often spoke of feeling vulnerable at work and sometimes when driving home, due to the risks and effects of (unwitting) second-hand exposures to NPS, especially when entering cells. Accounts of harms to themselves or colleagues because of involuntary inhalation of NPS as a bystander were described (Table 3b-4) and a few suggested the effects of NPS use in prison in general had significantly changed their work environment (Table 3b-5).

Value attached to e-cigarettes in prisons

E-cigarettes were viewed as having become an integral part of prison life over a relatively short space of time, serving similar functions to those previously fulfilled by tobacco, including as a valued commodity/form of prison currency. As with other 'alternative' currencies or valued items in prisons, this could create problems for staff and the prison system (as well as for PiC themselves).

Firstly, staff reported having to manage problems, including tensions in the halls and challenging behaviour when PiC ran out of e-liquids and wanted to borrow e-cigarette

For peer review only - http://bmjopen.bmj.com/site/about/guidelines.xhtml

For peer review only

products from others (Table 4a-1). Borrowing often came at a price; it was reportedly not uncommon for PiC to have to repay over the amount borrowed, leading to debt and in some cases bullying, in particular of more vulnerable PiC (Table 4a-2). One participant suggested that whilst issues of borrowing and debt (and bullying) were encountered in relation to other products, e-cigarette related debts could grow relatively quickly (Table 4a-3). Borrowing tobacco (before the smokefree policy was introduced) was seen as somewhat easier for PiC to manage, because it could be lent in small amounts, whereas borrowing e-cigarette products involved the loan of an e-liquid or device, both of higher monetary value.

Table 4: The value attached to e-cigarettes in prisons, and long term nicotine use and smoking cessation

4a The value attached to e-cigarettes in prisons

1. "…boys scratching the door, 'Oh boss, can you get us any vapes?' And then they're shouting to the other guys, 'Anyone got any?' and they were handed a capsule over with wee dribbles in it to keep them going." *C2-NV-NS*

2. "You have to try and nip that in the bud [vulnerable PiC getting into e-cigarette related debts], if you catch it, straight away." *G1-ExS-NV*

3. "There are, obviously, other things that get the guys in debt, but this seems to be the quick way...to borrow one [refill], pay back two, [and] not have the money [to buy your own] the following week." *G1-ExS-NV*

4. "We've already had an incident whereby one of the girls thought that she had caught something from the misuse of the vapes and that immediately stopped within the hall, for as far as we can try and control it." *B1-NS-NV*

5. "We had one [case] this morning actually. Someone was screaming and shouting because her vape has been nicked." *C1-CS-ExV*

For peer review only - http://bmjopen.bmj.com/site/about/guidelines.xhtml

4b Long-term nicotine use and maintained smoking cessation

1. "I think the smoking cessation works well, but I think the motivation for [some] prisoners to stop smoking disappears when they can have vapes. I think they were more motivated [to quit] just before the ban." *C3-NS-NV*

2. "They've purchased these vapes, bought them in jail, the first time they've taken them out with them [after release from prison] they've kept a hold of them because they're not got the financial money to go and buy cigarettes." *B1-NS-NV*

3. "Very seldom do I come across a prisoner who wants to stop smoking. Those who are being released keep telling me they're going over to the shop across the road to buy a pack of cigarettes when they get out. So they've got no intention of...the vape is just a short term thing for a lot of them." *G2-NS-NV*

4c Perceived potential solutions to the challenges raised by vaping in prisons

1. "because at the minute ….we're just giving it [e-cigarette products] out…there's nothing else other than 'vape, carry on vaping'." *G1-ExS-NV*

2. "Do the human rights of a prisoner outweigh the [human rights of the] guys who are working here?" *O3-NV-NS*

For peer review only

Secondly, the price of e-cigarette products was perceived to be a moderate or serious burden for some PiC, especially those on very low incomes. This sometimes led to the sharing of e-cigarette devices, and the exchange of part-used e-liquids (to enable use of different flavours at no additional cost) which raised concerns for staff about possible virus and disease transmission (Table 4a-4). (Note: interviews were conducted over a year before the first documented Covid-19 cases.)

For peer review only - http://bmjopen.bmj.com/site/about/guidelines.xhtml

There were several other ways that e-cigarettes created additional challenges for staff; for example, when PiC misplaced their device, the onus was on staff to break from their duties to locate it, and when e-cigarettes were reported as stolen (Table 4a-5). Several staff reported regular vaping in prohibited areas, and whilst some described this as unintentional, others reported instances where PiC had smuggled e-cigarettes outwith their cells, or had refused to go to appointments unless they were able to take their e-cigarettes with them. Some staff were willing to put PiC on report for repeated breaches, but others viewed the policing of e-cigarettes as too difficult because non-compliance was commonplace.

For peer review only

Long-term tobacco and nicotine use

Staff reported that prior to the introduction of e-cigarettes, some PiC had intended to become nicotine-free once smokefree rules were implemented. However, few PiC had reportedly achieved this; most previous smokers were vaping e-liquids containing nicotine long after tobacco had been removed from prisons (Table 4b-1). Staff were generally unsure about what support was available in prisons for PiC who wished to quit vaping and noted that e-liquids were only available in higher strengths in prisons, which some staff viewed as a potential significant barrier to enabling PiC to reduce nicotine dependence. While staff noted that some individuals might continue to vape exclusively after leaving prison, for example for financial reasons (Table 4b-2), several relayed discussions with PiC who intended to smoke tobacco on release, and in some cases this was considered a *fait accompli* (Table 4b-3), reflecting, for example, people's intentions to resume co-use of cannabis and tobacco.

Perceived potential solutions to the challenges raised by vaping in prisons

Participants expressed varied views on solutions to the perceived challenges of vaping in prisons. These included only selling single use e-cigarettes in prisons to limit product misuse; and supporting PiC to manage and, if desired, reduce use of rechargeable e-cigarettes (Table

For peer review only - http://bmjopen.bmj.com/site/about/guidelines.xhtml

4c-1). A few participants suggested e-cigarettes should be completely prohibited in prisons, with one suggesting this would better balance the 'rights' of staff with those of PiC (Table 4c-2). However, staff acknowledged that greater vaping restrictions were likely to be unpopular with PiC, and very challenging to implement given the scale and significance of vaping in prisons among PiC.

DISCUSSION

This study provides novel insights into prison staff experiences of the challenges associated with allowing e-cigarettes to be sold and used among PiC in smokefree prisons, alongside further evidence of perceived individual and organisational benefits of e-cigarettes in prisons. Findings suggest that staff were concerned, to varying degrees, by perceived negative impacts of e-cigarettes on worker health and safety, job tasks, and work environment. Given that the roles of prison staff are highly demanding [17], it is perhaps unsurprising that participants were very alert to potential risks associated with this major organisational change while generally expressing support, in national surveys, for having e-cigarettes in prisons for PiC.[5]

An area of concern to participants was the potential health impacts of (short and long term) exposure to e-cigarette vapour at work. These concerns are particularly understandable given that rates of vaping are around 10 times higher among PiC than the Scottish general population [18, 19] and that prison staff need to enter rooms where PiC regularly vape, to provide urgent care or maintain security. Prisons appear unusual among UK public sector organisations in permitting use of e-cigarettes indoors; a recent study found that vaping was prohibited indoors in all NHS Trusts and almost all Higher Education Institutions.[20] Differences in vaping policies between prisons and other workplaces may be viewed particularly negatively by prison staff (at a symbolic and/or practical level) given that the UK

For peer review only - http://bmjopen.bmj.com/site/about/guidelines.xhtml

prison workforce was exposed to SHS for over a decade longer than other UK workforces because prisons were partially exempt from Scotland's 2006 national smokefree laws. Concerns among prison staff in Scotland about the potential health effects of e-cigarettes on bystanders (and users) may also reflect broader anxieties about the safety of e-cigarettes; similar concerns have been expressed by other occupational groups e.g. healthcare staff.[21] Amongst the general population, a US study found that participants generally perceived e-cigarette vapour exposures to be '*moderately harmful'* to health; almost half believed that vaping should be prohibited in indoor public places such as restaurants.[22]

The use of e-cigarettes for illicit drug use is understandably of significant concern to prison staff given the adverse impacts that this can have on those living and working in prison and operational stability.[23] There is a growing body of evidence on the challenges and harms posed by NPS use in prisons, which had been identified as a challenge within the prisons several years prior to the introduction of smoke-free prison policy in Scotland.[24-26] Findings from TIPs interviews exploring post-implementation perspectives on Scotland's smoke-free prison policy amongst a separate sample of staff and people in custody also suggest that the smoke-free prison policy may have contributed to changes in the use of NPS, specifically through misuse of e-cigarettes.[6] Understandings of the general risks posed by e-cigarettes for illicit drug taking, including NPS, are developing: a 2018 systematic review [27] suggested several potential areas of risk. These include: that e-cigarettes might be viewed as a '*safer and innocuous method to experiment and try drugs*'; levels of illicit drug use might increase due to the ease with which e-cigarettes can be used for drug delivery; the emergence of new patterns of drug use; and illicit drug use may be easier to conceal, leading to increased problems with detection by authorities and greater risks of '*unintended or malicious'* exposures to bystanders (p107). It will be important to monitor these risks, including in prison settings. These findings highlight the ongoing challenges for prison

For peer review only - http://bmjopen.bmj.com/site/about/guidelines.xhtml

authorities to find appropriate e-cigarette devices for use in prisons, and other secure settings such as in-patient mental health services.

Prison staff also discussed challenges related to the value attached to e-cigarettes in prisons. These appeared similar to those previously caused by tobacco prior to smokefree prison policy and are to be expected, given prison rules on purchasing items from the prison shop and the informal economy in prisons.[28] (Analysis of canteen spend data on the relative costs of smoking and vaping in prisons is underway and will be reported separately.) Nonetheless, some staff might view such issues as particularly troublesome at a time when the workforce is facing wider challenges including increasing numbers of PiC, staff sickness absence rates and increased working hours.[29]

Finally, staff reported potential limitations or challenges of e-cigarettes for reducing tobacco harms in PiC long-term. Discomfort with continued use of nicotine among PiC may stem from staff knowledge that some individuals have a strong desire to become 'free' of substance dependence, as well as from (mis)understandings of health risks of nicotine [30] or perceptions that someone who vapes has not truly stopped 'smoking'.[31] Staff members' acknowledgement of risks that many PiC might resume smoking soon after leaving smokefree prisons, despite having exclusively vaped for a period, reflects the substantial challenges that people often face in transitioning from prison to the outside world.[32]

Findings reported here will be of interest to jurisdictions planning on making their prison systems smokefree and those considering use of e-cigarettes for harm reduction. They highlight the need for any policies on e-cigarette use in prisons to balance the needs of those PiC who wish to use e-cigarettes (in lieu of tobacco) against calls for precautionary measures to limit (or eliminate) e-cigarette vapour exposures because of a lack of long-term evidence on e-cigarette safety. Based on current evidence regarding their effects on air quality and

For peer review only - http://bmjopen.bmj.com/site/about/guidelines.xhtml

health [33], restricting indoor vaping in prisons to private rooms (cells) would appear proportionate, although there is a need for ongoing monitoring of the evidence and clear communication for the rationale for policies to prison staff. Like smoking previously, where allowed, vaping in prisons is likely to become normative and culturally embedded over time, potentially presenting future challenges for a prison service should the need to introduce tighter restrictions on e-cigarette use arise. Other challenges associated with the use of e-cigarettes among PiC might be reduced through providing guidance and support on appropriate use and on how to limit or quit use if desired (e.g., see practitioner guidance developed in Scotland, subsequent to these staff interviews, to support PiC to reduce or cease use of e-cigarettes [34]) and smoking relapse prevention interventions spanning the pre-post release period. Broader responses are also required to tackle illicit drug use among PiC, including measures to reduce environmental drivers of prison drug use.[35]

We believe the use of one-to-one interviews in this study is a strength because they enable in-depth exploration of people's perspectives and of confidential or sensitive issues [36]. Findings from this study complement and extend our previous work exploring staff perspectives on e-cigarettes using data gathered via focus groups before announcement of the smokefree policy,[11] and again post implementation.[6] A key study limitation is that certain participant groups (e.g. females, current vapers) may not be adequately represented in the sample, limiting our ability to explore potential between-group differences in perspectives. However, the inclusion of staff from a diverse set of prisons provides confidence that the breadth of challenges associated with e-cigarettes in prisons are likely to be reflected well in the data. It is important to note that data were collected prior to the introduction of guidance for practitioners on how to support PiC to reduce or stop using e-cigarettes and before the COVID-19 pandemic, both of which may have important implications for vaping in prisons and are priorities for future studies.

For peer review only - http://bmjopen.bmj.com/site/about/guidelines.xhtml

In conclusion, this detailed analysis, focused on the *challenges* associated with vaping in prisons from the perspective of prison staff, identified five main issues: staff concerns about exposures to e-cigarette vapour at work; use of e-cigarettes for illicit drug taking among PiC; the value attached to, and trading of, e-cigarette products in prisons; and implications for long-term nicotine and tobacco use. Addressing these challenges is likely to require a combination of measures specific to e-cigarettes and broader measures to address (illicit) drug use and promote overall health.

Acknowledgements

We are grateful to the prison staff who took part in the interviews, staff within the Scottish Prison Service who assisted with the study and facilitated access, and NHS staff who have supported our research on smoke-free prisons.

Contributors

KH, AB, HS and LB developed the study, with input from RO, DE, RP and the SPS Research Advisory Group. AB and KH liaised with the prison service and reported back on ongoing findings. AB prepared the ethics applications. RO, AB and DE conducted the qualitative interviews; all are highly experienced qualitative researchers. AB, RO and DM conducted analysis using the Framework approach. RO, AB and KH drafted the manuscript and all revisions. All other authors read a subsample of the transcripts and contributed to interpretation of the data and review of the final manuscript.

Ethical approval

Ethical approval was granted by the SPS Research Access and Ethics Committee (approval confirmed 23.8.18; no ref number) and by the University of Stirling General University Ethics Panel (ref: GUEP 497).

For peer review only - http://bmjopen.bmj.com/site/about/guidelines.xhtml

Data availability statement

No additional data available.

For peer review only

For peer review only - http://bmjopen.bmj.com/site/about/guidelines.xhtml

References

1 Brown A, O'Donnell R, Eadie D, et al. Initial Views and Experiences of Vaping in Prisons: A Qualitative Study With People in Custody Preparing for the Imminent Implementation of Scotland's Prison Smokefree Policy. *Nicotine Tob Res* 2021;23:543-549.

2 NHS Scotland. Smoke-free prisons pathway: A service specification supporting people in our care. 2018. http://www.healthscotland.scot/media/2276/smoke-free-prisons-pathway-a-service-specification-supporting-people-in-our-care.pdf (Accessed 24 September 2021).

3 Woodall J, Tattersfield A. Perspectives on implementing smoke-free prison policies in England and Wales. *Health Promot Int* 2018;33:1066-1073.

4 Kennedy SM, Davis SP, Thorne SL. Smoke-free policies in U.S. Prisons and jails: A review of the literature. *Nicotine Tob Res* 2015;17:629-635.

5 Sweeting H, Demou E, Brown A, et al. Prisoners and prison staff express increased support for prison smoking bans following implementation across Scotland: results from the Tobacco In Prisons study. *Tob Control* 2020;30:597-598.

6 Brown A, Mitchell D, Hunt K. Post-implementation perspectives on smokefree prison policy: a qualitative study with staff and people in custody. *Eur J Public Health* Published Online First: 27 August 2021. doi:10.1093/eurpub/ckab075/6358578

7 Beard E, West R, Michie S, et al. Association between electronic cigarette use and changes in quit attempts, success of quit attempts, use of smoking cessation pharmacotherapy, and use of stop smoking services in England: time series analysis of population trends. *BMJ* 2016;354:i4645.

8 Hartmann-Boyce J, McRobbie H, Lindson N, et al. Electronic cigarettes for smoking cessation. *Cochrane Database Syst Rev* 2020;10:CD010216. doi:10.1002/14651858.CD010216.pub4.

For peer review only - http://bmjopen.bmj.com/site/about/guidelines.xhtml

9 National Academies of Sciences Engineering and Medicine. Public Health Consequences of E-Cigarettes. Conclusions by Outcome. 2018. https://www.nap.edu/resource/24952/012318ecigaretteConclusionsbyOutcome.pdf (Accessed 24 September 2021).

10 Brown A, O'Donnell R, Eadie D, et al. E-cigarette Use in Prisons With Recently Established Smokefree Policies: A Qualitative Interview Study With People in Custody in Scotland. *Nicotine Tob Res* 2021;23:939-946.

11 Brown A, Sweeting H, Semple S, et al. Views of prison staff in Scotland on the potential benefits and risks of e-cigarettes in smoke-free prisons: a qualitative focus group study. *BMJ Open* 2019;9:e027799.

12 Curry L, Lee YO, Rogers T. E-cigarettes made especially for inmates. *Tob Control* 2014;23:e87-e88.

13 Young-Wolff KC, Karan LD, Prochaska JJ. Electronic cigarettes in jails: a panacea or public health problem? *JAMA Psychiatry* 2015;72:103-104.

14 Brown A, Sweeting H, Logan G, et al. Prison Staff and Prisoner Views on a Prison Smoking Ban: Evidence From the Tobacco in Prisons Study. *Nicotine Tob Res* 2018;21:1027-1035.

15 Spencer L RJ, O'Connor W, Morrell G, et al. Analysis in Practice. In: Ritchie J LJ, McNaughton Nicholls C, Ormston R, eds. Qualitative Research Practice. Sage, London: 2014:295-343.

16 Gale NK, Shapiro J, McLeod HS, et al. Patients-people-place: developing a framework for researching organizational culture during health service redesign and change. *Implement Sci* 2014;9:106.

17 Kinman G, Clements AJ, Hart J. Job demands, resources and mental health in UK prison officers. *Occup Med* 2017;67:456-460.

For peer review only - http://bmjopen.bmj.com/site/about/guidelines.xhtml

For peer review only

18 McLean J, Dean L. Scottish Health Survey 2018: Main report - Revised 2020. https://www.gov.scot/publications/scottish-health-survey-2018-volume-1-main-report/ (Accessed 24 September 2021).

19 Scottish Prison Service. 17th Prisoner Survery 2019. http://www.sps.gov.uk/nmsruntime/saveasdialog.aspx?fileName=17th+Prison+Survey+2019+-+Bulletin+Final7197_3445.pdf (Accessed 24 September 2021).

20 Blackwell AKM, Kosīte D, Marteau TM, et al. Policies for Tobacco and E-Cigarette Use: A Survey of All Higher Education Institutions and NHS Trusts in England. *Nicotine Tob Res* 2020;22:1235-1238.

21 Stepney M, Aveyard P, Begh R. GPs' and nurses' perceptions of electronic cigarettes in England: a qualitative interview study. *Br Journal Gen Pract* 2019;69:e8-e14.

22 Mello S, Bigman CA, Sanders-Jackson A, et al. Perceived Harm of Secondhand Electronic Cigarette Vapors and Policy Support to Restrict Public Vaping: Results From a National Survey of US Adults. *Nicotine Tob Res* 2016;18:686-693.

23 Kolind T, Duke K. Drugs in prisons: Exploring use, control, treatment and policy. *Drugs Educ Prev Pol* 2016;23:89-92.

24 Ralphs R, Williams L, Askew R, et al. The development of a synthetic cannabinoid market in an English prison. *Int J Drug Policy* 2017;40:57-69.

25 Corazza O, Coloccini S, Marrinan S, et al. Novel Psychoactive Substances in Custodial Settings: A Mixed Method Investigation on the Experiences of People in Prison and Professionals Working With Them. *Front Psychiatry* 2020;11:460.

26 Gooch K, Treadwell J. Prisoner Society in an Era of Psychoactive Substances, Organized Crime, New Drug Markets and Austerity. *B J Criminol* 2020;60:1260-1281.

27 Breitbarth AK, Morgan J, Jones AL. E-cigarettes—An unintended illicit drug delivery system. *Drug Alcohol Depend* 2018;192:98-111.

For peer review only - http://bmjopen.bmj.com/site/about/guidelines.xhtml

For peer review only

28 Walker DF. The informal economy in prison. *Crim Justice Matters* 2015;99:18-19.

29 Auditor General. The 2018/19 audit of the Scottish Prison Service. 2020 https://www.audit-scotland.gov.uk/uploads/docs/report/2019/s22_190912_sps.pdf (Accessed 24 September 2021).

30 O'Brien EK, Nguyen AB, Persoskie A, et al. U.S. adults' addiction and harm beliefs about nicotine and low nicotine cigarettes. *Prev Med* 2017;96:94-100.

31 Hartwell G, Egan M, Petticrew M. Understanding decisions to use e-cigarettes or behavioural support to quit tobacco: a qualitative study of current and ex-smokers and stop smoking service staff. *Addiction* 2020;115:518-526.

32 Puljevic C, Segan CJ. Systematic Review of Factors Influencing Smoking Following Release From Smoke-Free Prisons. *Nicotine Tob Res* 2019;21:1011-1020.

33 Li L, Lin Y, Xia T, et al. Effects of Electronic Cigarettes on Indoor Air Quality and Health. *Annu Rev Public Health* 2020;41:363-380.

34 NHS Health Scotland. Supporting individuals to cut down and quit vaping: Guidance for NHS Quit Your Way advisers based in prisons. 2020. http://www.healthscotland.scot/media/2955/supporting-individuals-to-cut-down-and-quit-vaping.pdf (Accessed 24 September 2021).

35 Mjåland K. Exploring prison drug use in the context of prison-based drug rehabilitation. *Drugs Educ Prev Pol* 2016;23:154-162.

36 Guest G, Namey E, Mitchell M. In-depth interviews. *Collecting Qualitative Data*: SAGE Publications, Ltd. 2013:113-171.

For peer review only

COREQ (COnsolidated criteria for REporting Qualitative research) Checklist

A checklist of items that should be included in reports of qualitative research. You must report the page number in your manuscript where you consider each of the items listed in this checklist. If you have not included this information, either revise your manuscript accordingly before submitting or note N/A.

Topic	Item No.	Guide Questions/Description	Reported on Page No.
**Domain 1: Research team and reflexivity**			
Personal characteristics			
Interviewer/facilitator	1	Which author/s conducted the interview or focus group?	
Credentials	2	What were the researcher's credentials? E.g. PhD, MD	
Occupation	3	What was their occupation at the time of the study?	
Gender	4	Was the researcher male or female?	
Experience and training	5	What experience or training did the researcher have?	
Relationship with participants			
Relationship established	6	Was a relationship established prior to study commencement?	
Participant knowledge of the interviewer	7	What did the participants know about the researcher? e.g. personal goals, reasons for doing the research	
Interviewer characteristics	8	What characteristics were reported about the inter viewer/facilitator? e.g. Bias, assumptions, reasons and interests in the research topic	
**Domain 2: Study design**			
Theoretical framework			
Methodological orientation and Theory	9	What methodological orientation was stated to underpin the study? e.g. grounded theory, discourse analysis, ethnography, phenomenology, content analysis	
Participant selection			
Sampling	10	How were participants selected? e.g. purposive, convenience, consecutive, snowball	
Method of approach	11	How were participants approached? e.g. face-to-face, telephone, mail, email	
Sample size	12	How many participants were in the study?	
Non-participation	13	How many people refused to participate or dropped out? Reasons?	
Setting			
Setting of data collection	14	Where was the data collected? e.g. home, clinic, workplace	
Presence of non-participants	15	Was anyone else present besides the participants and researchers?	
Description of sample	16	What are the important characteristics of the sample? e.g. demographic data, date	
Data collection			
Interview guide	17	Were questions, prompts, guides provided by the authors? Was it pilot tested?	
Repeat interviews	18	Were repeat inter views carried out? If yes, how many?	
Audio/visual recording	19	Did the research use audio or visual recording to collect the data?	
Field notes	20	Were field notes made during and/or after the inter view or focus group?	
Duration	21	What was the duration of the inter views or focus group?	
Data saturation	22	Was data saturation discussed?	
Transcripts returned	23	Were transcripts returned to participants for comment and/or	

For peer review only

Topic	Item No.	Guide Questions/Description	Reported on Page No.
		correction?	
**Domain 3: analysis and findings**			
Data analysis			
Number of data coders	24	How many data coders coded the data?	
Description of the coding tree	25	Did authors provide a description of the coding tree?	
Derivation of themes	26	Were themes identified in advance or derived from the data?	
Software	27	What software, if applicable, was used to manage the data?	
Participant checking	28	Did participants provide feedback on the findings?	
Reporting			
Quotations presented	29	Were participant quotations presented to illustrate the themes/findings? Was each quotation identified? e.g. participant number	
Data and findings consistent	30	Was there consistency between the data presented and the findings?	
Clarity of major themes	31	Were major themes clearly presented in the findings?	
Clarity of minor themes	32	Is there a description of diverse cases or discussion of minor themes?	

Developed from: Tong A, Sainsbury P, Craig J. Consolidated criteria for reporting qualitative research (COREQ): a 32-item checklist for interviews and focus groups. *International Journal for Quality in Health Care*. 2007. Volume 19, Number 6: pp. 349 – 357

Once you have completed this checklist, please save a copy and upload it as part of your submission. DO NOT include this checklist as part of the main manuscript document. It must be uploaded as a separate file.

For peer review only

BMJ Open

The challenges associated with e-cigarette use by people in custody in Scottish prisons: a qualitative interview study with prison staff

Journal:	*BMJ Open*
Manuscript ID	bmjopen-2021-051009.R2
Article Type:	Original research
Date Submitted by the Author:	14-Dec-2021
Complete List of Authors:	O'Donnell, Rachel; University of Stirling, Institute of Social Marketing and Health<br>Brown, Ashley; University of Stirling Institute for Social Marketing<br>Eadie, Douglas; Institute for Social Marketing, School of Health Sciences<br>Mitchell, Danielle; Institute for Social Marketing and Health, University of Stirling, Faculty of Health Sciences and Sport<br>Bauld, Linda; University of Edinburgh, Usher Institute and UK Centre for Tobacco and Alcohol Studies, College of Medicine and Veterinary Medicine<br>Demou, Evangelia; University of Glasgow, MRC/CSO Social & Public Health Sciences Unit<br>Purves, Richard; University of Stirling, Institute for Social Marketing and Health<br>Sweeting , Helen ; University of Glasgow, MRC/CSO Social & Public Health Sciences Unit<br>Hunt, Kate; University of Stirling Institute for Social Marketing and Health, Institute for Social Marketing and Health
**Primary Subject Heading**:	Public health
Secondary Subject Heading:	Smoking and tobacco
Keywords:	PUBLIC HEALTH, QUALITATIVE RESEARCH, OCCUPATIONAL & INDUSTRIAL MEDICINE

SCHOLARONE™
Manuscripts

I, the Submitting Author has the right to grant and does grant on behalf of all authors of the Work (as defined in the below author licence), an exclusive licence and/or a non-exclusive licence for contributions from authors who are: i) UK Crown employees; ii) where BMJ has agreed a CC-BY licence shall apply, and/or iii) in accordance with the terms applicable for US Federal Government officers or employees acting as part of their official duties; on a worldwide, perpetual, irrevocable, royalty-free basis to BMJ Publishing Group Ltd ("BMJ") its licensees and where the relevant Journal is co-owned by BMJ to the co-owners of the Journal, to publish the Work in this journal and any other BMJ products and to exploit all rights, as set out in our licence.

The Submitting Author accepts and understands that any supply made under these terms is made by BMJ to the Submitting Author unless you are acting as an employee on behalf of your employer or a postgraduate student of an affiliated institution which is paying any applicable article publishing charge ("APC") for Open Access articles. Where the Submitting Author wishes to make the Work available on an Open Access basis (and intends to pay the relevant APC), the terms of reuse of such Open Access shall be governed by a Creative Commons licence – details of these licences and which Creative Commons licence will apply to this Work are set out in our licence referred to above.

Other than as permitted in any relevant BMJ Author's Self Archiving Policies, I confirm this Work has not been accepted for publication elsewhere, is not being considered for publication elsewhere and does not duplicate material already published. I confirm all authors consent to publication of this Work and authorise the granting of this licence.

For peer review only

The challenges associated with e-cigarette use by people in custody in Scottish prisons: a qualitative interview study with prison staff

Rachel O'Donnell[1*], Ashley Brown[1*], Douglas Eadie[1], Danielle Mitchell[1], Linda Bauld[2], Evangelia Demou[3], Richard Purves[1], Helen Sweeting[3] and Kate Hunt[1]

1. Institute for Social Marketing and Health, Faculty of Health Sciences and Sport, University of Stirling, Pathfoot Building, Stirling, FK9 4LA.

2. Usher Institute and SPECTRUM Consortium, College of Medicine and Veterinary Medicine, University of Edinburgh.

3. MRC/CSO Social and Public Health Sciences Unit, Berkeley Square, 99 Berkeley Street, Glasgow, G3 7HR.

* RO and AB are joint first authors and contributed equally to this work.

Corresponding author: Rachel O'Donnell r.c.odonnell@stir.ac.uk

For peer review only

Word Count: 3829/4000 words

Keywords: e-cigarettes, vaping, prisoner health, staff, workplace, qualitative

ABSTRACT

Objectives: Little is known about the perspectives of staff working in prisons where e-cigarettes are permitted. Scotland now permits people in custody (PiC), but not staff/visitors to use e-cigarettes, following implementation of smoke-free prisons policy in 2018. Previous studies, conducted before and immediately after the introduction of e-cigarettes in Scottish prisons, have evidenced stakeholder support for their use by PiC. This study focuses on key challenges associated with e-cigarette use in prisons, using data collected from prison staff once e-cigarettes had been allowed in a smoke-free environment for 6-9 months.

Setting: Five prisons in Scotland.

Participants: Sixteen qualitative interviews were conducted with prison staff from five prisons varying by population (sex, age and sentence length). Data were managed and analysed using the Framework approach.

Results: Whilst these staff confirmed strong support for the smoke-free prison policy and reported some benefits of replacing tobacco with e-cigarettes, they also spoke of the challenges e-cigarettes pose. These included: workplace e-cigarette vapour exposures; perceptions that e-cigarettes provide a new, effective way for some PiC to take illegal drugs, particularly new psychoactive substances; organisational challenges relating to the value attached to e-cigarettes in prisons; and implications for long-term nicotine use and tobacco cessation. Staff anticipated difficulties in tightening restrictions on e-cigarette use by PiC given its scale and significance among this population.

Conclusions: Maximizing the benefits of e-cigarette use by PiC is likely to require multiple measures to support effective and safe use and e-cigarette reduction/cessation where desired. This includes monitoring any misuse of e-cigarettes, and provision of guidance and support on appropriate e-cigarette use and how to limit or quit use if desired. Findings are relevant to jurisdictions considering or planning changes in prison smoking or vaping policies.

For peer review only - http://bmjopen.bmj.com/site/about/guidelines.xhtml

Strengths and limitations of the study

- Conducting interviews with prison staff in a private room facilitated open discussions about their experiences of the challenges associated with e-cigarette use among people in custody in Scottish prisons.
- The use of a qualitative approach in this study broadens understanding of risks and challenges of use of e-cigarettes in this distinct setting and corresponding measures that might minimise harms and maximise benefits.
- Whilst some staff groups (e.g. female members of staff, current vapers) may not be adequately represented in the sample, the inclusion of staff from a diverse set of prisons provides confidence that the breadth of challenges associated with e-cigarettes in prisons are likely to be reflected.

Funding statement: This work was supported by Cancer Research UK (C45874/A27016). ED and HS were also supported by the UK Medical Research Council MC_UU_12017/12 and Scottish Chief Scientist Office SPHSU12.

Competing interests statement: None to declare

For peer review only - http://bmjopen.bmj.com/site/about/guidelines.xhtml

INTRODUCTION

In anticipation of the November 2018 implementation of the country's comprehensive (indoor/outdoor) smoke-free prison policy, legislative changes occurred which enabled people in custody (PiC) in Scotland to purchase and use single-use and rechargeable e-cigarettes in rooms (cells) and outdoor spaces from September 2018. The places where e-cigarette use is permitted mirrors the places where PiC were allowed to smoke tobacco prior to implementation of the smoke-free policy (i.e. in designated rooms and in some outdoor spaces). Tobacco stopped being sold in prisons via the prison shop ('canteen') ~two weeks before the smoke-free policy was introduced. Prison staff and visitors remained prohibited from smoking, or using e-cigarettes (hereafter vaping), on prison property. Information on e-cigarette products sold via the canteen in Scottish prisons and prison smoking cessation services is available elsewhere.(1, 2) The introduction of e-cigarettes in prisons in Scotland (and in other jurisdictions such as England and Wales, and the US (3, 4)) has potentially important implications for the health and safety of PiC and prison staff, and the operation of prison systems. There is very limited evidence on e-cigarette use in prisons, with no studies reporting on the perspectives of staff working in prisons where vaping is permitted among PiC, thus limiting understandings of its perceived benefits and risks in this distinct setting.

Current research evidence on e-cigarette use in prisons comes from two studies of tobacco and vaping in Scottish prisons (the Tobacco in Prisons study (TIP) and the E-cigarettes in Prisons study).(1, 5-7) Evidence from TIPs, and responses to a public consultation on smoke-free prisons (by the Scottish Prison Service)(8), showed that e-cigarettes received broad support from stakeholders in anticipation of the introduction of smoke-free prison policy. For example, TIPs surveys in May-June 2018 (after the smoke-free policy announcement but before it was known e-cigarettes would be made available to PiC) and May-June 2019 (6-9 months after use of (rechargeable) e-cigarettes was first allowed and smoke-free policy was

For peer review only - http://bmjopen.bmj.com/site/about/guidelines.xhtml

implemented in Scottish prisons) both showed the majority of prison staff (~75%) and almost all PiC (~90%) agreed that 'e-cigarettes should be available to help prisoners to stop smoking/manage without tobacco'.(5)

Complementary qualitative research suggests this broad support for e-cigarettes in Scottish prisons has been substantially influenced by perceptions that they are beneficial in helping PiC and staff to cope with the major operational change represented by the removal of tobacco.(9) Favourable perceptions of e-cigarettes in Scottish prisons also reflect broader evidence on their appeal and effectiveness for some current and ex-smokers (e.g. those who feel unwilling or unable to quit nicotine)(10, 11) and evidence on reduced harms to users and bystanders from vaping relative to smoking.(12)

However, important issues and challenges in relation to permitting PiC to vape were also voiced by prison staff and some PiC in anticipation of their introduction,(1, 13) as well as by commentators in other jurisdictions.(14, 15) These concerns relate to e-cigarette safety, implications for long-term tobacco and nicotine cessation (e.g. uncertainties about whether e-cigarette use in prison increases or decreases the likelihood of return to smoking on release), and risks associated with vaping related to unique features of prison environments. These include: rules around earnings and purchases that mean PiC are not always able to (formally) buy (enough of) the items they want or 'need'; operation of illicit prison economies in which any sought-after item can be traded or 'bought' using alternative currencies; and well established practices of PiC repurposing items, including for illicit purposes such as drug taking.(1) The benefits of e-cigarettes in prisons are well covered in our previous publications (including focus groups with prison staff before rules were changed to allow PiC to vape;(7, 13) interviews with PiC immediately after e-cigarettes were introduced (1) and again once new rules on e-cigarettes and smoking had bedded in;(16) and repeat cross sectional surveys of prison staff and PiC(5)). This paper therefore focuses on the *challenges* of permitting the

For peer review only - http://bmjopen.bmj.com/site/about/guidelines.xhtml

use of e-cigarettes in prisons identified by prison staff, based on novel interview data collected after vaping in prisons had become well established (8-11 months after sales of rechargeable e-cigarettes first began in Scottish prisons and 6-9 months after smoke-free rules were implemented). Together with our previous studies, the current findings will be relevant for prison systems (and institutions such as mental health inpatient services) seeking to implement, and maximise the benefits of, smoke-free policies.

METHODS

Patient and Public Involvement

The study was designed to ensure that the views of prison staff could be heard at different stages of the process of implementing smoke-free prisons in Scotland. A Research Advisory Group which included representation from prison management and union members provided feedback on the overall design of the study, study materials, and early findings to inform data collection and interpretation. Staff within the prisons were involved in the dissemination of information about the study and recruitment to the study. Personnel involved in implementing and managing smoke-free policies were also involved in discussion of findings to establish recommendations for future policymakers and implementers.

Sample and recruitment

Data were collected between May-August 2019 from 16 Scottish Prison Service staff working in five (of Scotland's 15) prisons which varied by prison population (sex, age and sentence length) and capacity. Participants were recruited through designated staff contacts who were asked to invite staff from a range of work roles (e.g. residential; admissions; health and safety; prison management), to enable the exploration of diverse views. Our contacts

For peer review only

scheduled one-to-one meetings between researchers and staff who expressed interest in participation. Sample characteristics are detailed in Table 1.

Table 1: Participant Characteristics (n=16)

**Work role**		**Number of years of service**	
Residential officer	8	1-10	2
Non-residential officer	6	11-20	6
Managerial	1	21-30	5
Missing	1	31-40	2
		Missing	1
**Sex**		**Age (years)**	
Male	13	18-30	2
Female	3	31-40	2
		41-50	3
		51-60	8
		Missing	1
**Smoking status**		**Vaping status**	
Non smoker (NS)	9	Non vaper (NV)	11
Ex smoker (ExS)	4	Ex vaper (ExV)	3
Current smoker (CS)	2	Current vaper (CV)	1
Missing	1	Missing	1

Data collection

Interviews (averaging 35 minutes) were conducted in a room at the prison which allowed the participant to speak freely. After providing information about the study and answering any

For peer review only - http://bmjopen.bmj.com/site/about/guidelines.xhtml

questions, written or audio-recorded verbal consent was obtained from each participant by one of three interviewers (RO, AB, DE) before s/he commenced the interview. The topic guide covered: participant background, smoking and vaping history; views and experiences related to vaping in the prison context; and views on the positive and negative consequences of vaping by PiC for prison staff, the prison regime and PiC themselves. Question wording and order were varied as appropriate and participants were invited to raise any additional points they considered important.

Data analysis

Fifteen of the 16 interviews were audio-recorded and transcribed by a professional transcription agency; detailed notes were made in lieu of audio-recording of one interview on participant request. All interview content was de-identified, then analysed (by RO, AB, DM) using the Framework approach.(17) This approach is well suited to the analysis of in-depth interview data, as summarisation of data into a framework grid within the NVivo program facilitates analysis across cases without losing the wider context of each participant's account.(18) A thematic framework was developed (by AB, RO) to guide data management and analysis, based on deductive (considering the topic guide, relevant literature) and inductive (reading transcripts) techniques. In preparation for detailed analysis, data summaries were written in relevant cells of the framework grid (RO, DM, AB), incorporating hyperlinks to transcripts to facilitate data retrieval. RO reviewed all summaries to check data interpretation and consistency of approach. Data summaries were then used to identify high level themes (AB, RO) before further in-depth analysis was conducted (RO), focusing on data about challenges posed by e-cigarettes in prisons. RO and AB finalised themes based on re-examining data and reflexive team discussions. Themes are presented below alongside illustrative quotes in Tables 2, 3 and 4; each quote indicates the prison the participant worked in, their interview ID number and smoking and vaping status.

For peer review only - http://bmjopen.bmj.com/site/about/guidelines.xhtml

For peer review only

RESULTS

Staff perceptions of the smoke-free prison policy and e-cigarette availability for PiC

Prior to reporting staff perspectives of the *challenges* of permitting the use of e-cigarettes in prisons, we briefly summarise, for context, their opinions on smoke-free policy and beliefs about the benefits of e-cigarette availability in prisons. Staff interviewed for this study voiced strong overall support for the smoke-free prison policy and reported positive impacts of removing tobacco from prisons, including benefits for their own health and comfort while at work (Table 2-1) (see also (1, 13)). E-cigarettes were generally believed to have supported smoke-free prison policy implementation by helping PiC to manage without tobacco (Table 2-2). Benefits for reducing tobacco-related harms among PiC and staff were sometimes discussed. However, whilst most participants acknowledged some benefits associated with the introduction of e-cigarettes in prisons, several drawbacks were also discussed, leading some to reflect on whether smoke-free prisons could have been achieved without allowing PiC to vape. For example, some staff suggested in retrospect that the service might have been able to manage any temporary disruption that could have arisen from the removal of tobacco from prisons (Table 2, 3-4).

Table 2: Staff perceptions of the smoke-free prison policy and e-cigarette availability

1. "It's night and day in that the smell of tobacco's not there. You're not breathing it in. …it's not in your clothes. You're not bringing it home to your family. That to me is a big thing." *L4-NS-NV*
2. "The fact that the e-cigarettes gave an alternative to the cigarettes actually made the transition of the jail changing over to no smoking a lot easier…it was a massive help to us." *B1-NS-NV*

For peer review only - http://bmjopen.bmj.com/site/about/guidelines.xhtml

3	"…making jails smoke-free, we could just say, right that's it [no nicotine substitute], and we would have had bother [from PiC]. But would we have got through that bother, and then not had the smokers [vapers]?" *C2-NS-NV*
4	"The vapes made the process from smoking tobacco to there not being tobacco, it made that an easy process because we gave the prisoners a new option, while we kind of said to ourselves…'Why not just take the hit [i.e. without introducing e-cigarettes] and see how it goes with no tobacco?' Would that not have been better? Especially because I don't think there's enough research into the e-cigarettes and the vaping as well. We don't know exactly what kind of consequences there are." *O3-NS-NV*

Staff exposures to e-cigarette vapour

Several participants expressed doubts about the safety of vaping for themselves as bystanders (Table3a-1), in many cases referring to the absence of long-term studies on exposure to e-cigarette vapour (Table 3a-2). Concerns about exposure to e-cigarette vapour whilst at work were discussed in the context of very widespread vaping among PiC (Table 3a-3). In this context, some staff reported practical challenges in avoiding exposures to e-cigarette vapour when carrying out tasks such as cell searches (Table 3a-4). One participant wondered whether the persistent cough she had started to experience was linked to workplace exposure to e-cigarette vapour.

Table 3: Staff exposures to e-cigarette vapour, and misuse of e-cigarettes among PiC to take illegal drugs

**3a) Staff exposures to e-cigarette vapour**
1. "I wonder what the harm is of passive smoking with the vapes. I know we've taken away a lot of chemicals and tar and things that were in tobacco and that's great but…what is in the actual oils [e-liquids] and is that potentially doing harm? *C1-CS-EV*

For peer review only - http://bmjopen.bmj.com/site/about/guidelines.xhtml

2. Am I any better off? We won't know for 20 years." C2-NS-NV

3. "I would say ninety-five per cent of them use e-cigarettes, ninety percent or more..." *G2–NS-NV*"

4. "If you went to somebody's cell and it's a spontaneous job and somebody's vaping in the cell, how realistic is it for you to say…'You need to stop vaping! You need to stop that for an hour before I come into this cell'? It's impossible." *O3-NS-NV*

3b) Misuse of e-cigarettes to take illegal drugs

1. "There has been a lot of instances of vapes being broken, or improvised, shall we say …prisoners now know they can put paper in. It's obviously soaked in NPS (novel psychoactive substances)…and that's now their hit." *L3-NS-NV*

2. "The thing that prisoners have got is time to figure out how to take drugs…not every prisoner is like that obviously. It's only a certain amount that smoke drugs…but they adapt a different way of doing it and the thing is, they've got time to figure that out." *O3-NS-NV*

3. "The vape's just good [for NPS use] because it's enclosed… and also how do you police 500, 400 and odd people, in some cases the bigger jails, you've got a thousand odd prisoners. How do you police every single person with a vape?" *B1-NS-NV*

4. "A lot of staff become ill because they've entered somebody's cell to speak to them and by the time they've left they've started to feel…its [second-hand exposure to NPS] taken effect. So I'm quite conscious if I go to a cell. If it's smoky [filled with e-cigarette vapour], I'm not so keen to go in there…it feels like it's a matter of time given how many of my colleagues have been affected by it…Headaches, high blood pressure, heartrate zooming up, becoming shaky and unsteady. Staff have been really, really poorly with it." *C1-CS-ExV*

For peer review only - http://bmjopen.bmj.com/site/about/guidelines.xhtml

5. "It's a completely different workplace now, and this is definitely something new for me, in the years I've worked here I've never known this before." *O4-ExS-ExV*

Misuse of e-cigarettes among PiC to take illegal drugs

Concerns about the risks associated with exposure to e-cigarette vapour were amplified because participants in all prisons viewed (rechargeable) e-cigarettes as providing a new and effective way for some PiC to take NPS (Table 3b-1), particularly since some PiC had a lot of unoccupied time that could be spent on (devising) illicit activity (Table 3b-2). Whilst some noted the removal of lighters as part of smoke-free policy may have contributed to increased NPS use (replacing cannabis which PiC were less able to ignite), one reported that the number of PiC being placed on observations for drug use had increased over several years and so current NPS use might only reflect an underlying trend.

Staff working in residential areas of prisons gave accounts of how some PiC adapted e-cigarette devices to enable the ingestion of vapour from NPS-soaked paper and noted the unpredictable and adverse effects of this practice on PiC. Residential staff also noted that NPS use was easily concealed within an e-cigarette, and often odourless, adding to concerns about 'policing' NPS use (Table 3b-3). They often spoke of feeling vulnerable at work and sometimes when driving home, due to the risks and effects of (unwitting) second-hand exposures to NPS, especially when entering cells. Accounts of harms to themselves or colleagues because of involuntary inhalation of NPS as a bystander were described (Table 3b-4) and a few suggested the effects of NPS use in prison in general had significantly changed their work environment, for example by undermining safety and security for staff and PiC (Table 3b-5).

Value attached to e-cigarettes in prisons

For peer review only - http://bmjopen.bmj.com/site/about/guidelines.xhtml

E-cigarettes were viewed as having become an integral part of prison life over a relatively short space of time, serving similar functions to those previously fulfilled by tobacco, including as a valued commodity/form of prison currency. As with other 'alternative' currencies or valued items in prisons, this could create problems for staff and the prison system (as well as for PiC themselves).

Firstly, staff reported having to manage problems, including tensions in the halls and challenging behaviour when PiC ran out of e-liquids and wanted to borrow e-cigarette products from others (Table 4a-1). Borrowing often came at a price; it was reportedly not uncommon for PiC to have to repay over the amount borrowed, leading to debt and in some cases bullying, in particular of more vulnerable PiC (Table 4a-2). One participant suggested that whilst issues of borrowing and debt (and bullying) were encountered in relation to other products, e-cigarette related debts could grow relatively quickly (Table 4a-3). Borrowing tobacco (before the smoke-free policy was introduced) was seen as somewhat easier for PiC to manage, because it could be lent in small amounts, whereas borrowing e-cigarette products involved the loan of an e-liquid or device.

Table 4: The value attached to e-cigarettes in prisons, and long term nicotine use and smoking cessation

4a The value attached to e-cigarettes in prisons

1. "…boys scratching the door, 'Oh boss, can you get us any vapes?' And then they're shouting to the other guys, 'Anyone got any?' and they were handed a capsule over with wee dribbles in it to keep them going." *C2-NV-NS*

2. "You have to try and nip that in the bud [vulnerable PiC getting into e-cigarette related debts], if you catch it, straight away." *G1-ExS-NV*

For peer review only

3. "There are, obviously, other things that get the guys in debt, but this seems to be the quick way...to borrow one [refill], pay back two, [and] not have the money [to buy your own] the following week." *G1-ExS-NV*

4. "We've already had an incident whereby one of the girls thought that she had caught something from the misuse of the vapes and that immediately stopped within the hall, for as far as we can try and control it." *B1-NS-NV*

5. "We had one [case] this morning actually. Someone was screaming and shouting because her vape has been nicked." *C1-CS-ExV*

4b Long-term nicotine use and maintained smoking cessation

1. "I think the smoking cessation works well, but I think the motivation for [some] prisoners to stop smoking disappears when they can have vapes. I think they were more motivated [to quit smoking] just before the ban." *C3-NS-NV*

2. "They've purchased these vapes, bought them in jail, the first time they've taken them out with them [after release from prison] they've kept a hold of them because they've not got the financial money to go and buy cigarettes." *B1-NS-NV*

3. "Very seldom do I come across a prisoner who wants to stop smoking. Those who are being released keep telling me they're going over to the shop across the road to buy a pack of cigarettes when they get out. So they've got no intention of...the vape is just a short term thing for a lot of them." *G2-NS-NV*

4c Perceived potential solutions to the challenges raised by vaping in prisons

1. "There's no way you can, you know, manipulate that [single-use] device to [take NPS]…and that's why a lot of the staff are asking that they bring that in." *B1-NS-NV*

2. "We've gone from smoking to vaping, are we just maintaining now, or do we now have to start looking at having a reduction programme for the vapers?" *C2-NV-NS*

For peer review only - http://bmjopen.bmj.com/site/about/guidelines.xhtml

3. “Do the human rights of a prisoner outweigh the [human rights of the] guys who are working here?” *O3-NV-NS*

Secondly, the price of e-cigarette products was perceived to be a moderate or serious burden for some PiC, especially those on very low incomes, as tobacco had been for some PiC before the smoke-free rules. This sometimes led to the sharing of e-cigarette devices, and the exchange of part-used e-liquids (to enable use of different flavours at no additional cost) which raised concerns for staff about possible virus and disease transmission (Table 4a-4). (Note: interviews were conducted over a year before the first documented COVID-19 cases.)

There were several other ways that e-cigarettes created additional challenges for staff; for example, when PiC misplaced their device, the onus was on staff to break from their duties to locate it, and when e-cigarettes were reported as stolen (Table 4a-5). Several staff reported regular vaping in prohibited areas (e.g. vaping outside designated rooms and some outdoor spaces), and whilst some described this as unintentional, others reported instances where PiC had deliberately hidden e-cigarettes whilst moving through areas of the prison where vaping was not allowed, or had refused to go to appointments unless they were able to take their e-cigarettes with them. Some staff were willing to put PiC on report for repeated breaches, but others viewed the policing of e-cigarettes as too difficult because non-compliance was commonplace.

Long-term tobacco and nicotine use

Staff reported that prior to the introduction of e-cigarettes, some PiC had intended to become nicotine-free once smoke-free rules were implemented. However, few PiC had reportedly achieved this; most previous smokers were vaping e-liquids containing nicotine long after tobacco had been removed from prisons (Table 4b-1). Staff were generally unsure about

For peer review only - http://bmjopen.bmj.com/site/about/guidelines.xhtml

what support was available in prisons for PiC who wished to quit vaping and noted that e-liquids were only available in higher strengths in prisons, which some staff viewed as a potential significant barrier to enabling PiC to reduce use of e-cigarettes/nicotine dependence. While staff noted that some individuals might continue to vape exclusively after leaving prison, for example for financial reasons (Table 4b-2), several relayed discussions with PiC who intended to smoke tobacco on release, and in some cases this was considered a *fait accompli* (Table 4b-3), reflecting, for example, people's intentions to resume co-use of cannabis and tobacco.

Perceived potential solutions to the challenges raised by vaping in prisons

Participants expressed varied views on solutions to the perceived challenges of vaping in prisons. These included only selling reduced strength e-cigarettes in prisons to limit product misuse; and supporting PiC to manage and, if desired, reduce use of rechargeable e-cigarettes (Table 4c-1). A few participants suggested e-cigarettes should be completely prohibited in prisons, with one suggesting this would better balance the 'rights' of staff with those of PiC (Table 4c-2). However, staff acknowledged that greater vaping restrictions were likely to be unpopular with PiC, and very challenging to implement given the scale and significance of vaping in prisons among PiC.

DISCUSSION

This study provides novel insights into prison staff experiences of the challenges associated with allowing e-cigarettes to be sold and used among PiC in smoke-free prisons, alongside further evidence of perceived individual and organisational benefits of e-cigarettes in prisons. Findings suggest that staff were concerned, to varying degrees, by perceived negative impacts of e-cigarettes on worker health and safety, job tasks, and work environment. Given that the roles of prison staff are highly demanding (19), it is perhaps unsurprising that participants

For peer review only - http://bmjopen.bmj.com/site/about/guidelines.xhtml

were very alert to potential risks associated with this major organisational change while generally expressing support, in national surveys, for having e-cigarettes in prisons for PiC.(5)

An area of concern to participants was the potential health impacts of (short and long term) exposure to e-cigarette vapour at work. These concerns are particularly understandable given that rates of vaping are around 10 times higher among PiC than the Scottish general population (20, 21) and that prison staff need to enter rooms where PiC regularly vape, to provide urgent care or maintain security. Prisons appear unusual among UK public sector organisations in permitting use of e-cigarettes indoors; a recent study found that vaping was prohibited indoors in all NHS Trusts and almost all Higher Education Institutions.(22) Differences in vaping policies between prisons and other workplaces may be viewed particularly negatively by prison staff given that the UK prison workforce was exposed to second-hand smoke for over a decade longer than other UK workforces because prisons were partially exempt from Scotland's 2006 national smoke-free laws. Concerns among prison staff in Scotland about the potential health effects of e-cigarettes on bystanders (and users) may also reflect broader anxieties about the safety of e-cigarettes; similar concerns have been expressed by other occupational groups e.g. healthcare staff.(23) Amongst the general population, a US study found that participants generally perceived e-cigarette vapour exposures to be '*moderately harmful'* to health; almost half believed that vaping should be prohibited in indoor public places such as restaurants.(24)

The use of e-cigarettes for illicit drug use is understandably of significant concern to prison staff given the adverse impacts that this can have on those living and working in prison and operational stability.(25) There is a growing body of evidence on the challenges and harms posed by NPS use in prisons, which had been identified as a challenge within the prisons several years prior to the introduction of smoke-free prison policy in Scotland.(26-28)

For peer review only

Findings from TIPs interviews exploring post-implementation perspectives on Scotland's smoke-free prison policy amongst a separate sample of staff and people in custody also suggest that the smoke-free prison policy may have contributed to changes in the use of NPS, specifically through misuse of e-cigarettes.(9) Understandings of the general risks posed by e-cigarettes for illicit drug taking, including NPS, are developing: a 2018 systematic review (29) suggested several potential areas of risk. These include: that e-cigarettes might be viewed as a '*safer and innocuous method to experiment and try drugs*'; levels of illicit drug use might increase due to the ease with which e-cigarettes can be used for drug delivery; the emergence of new patterns of drug use; and illicit drug use may be easier to conceal, leading to increased problems with detection by authorities and greater risks of '*unintended or malicious'* exposures to bystanders (p107). It will be important to monitor these risks, including in prison settings. These findings highlight the ongoing challenges for prison authorities to find appropriate e-cigarette devices for use in prisons, and other secure settings such as in-patient mental health services.

Prison staff also discussed challenges related to the value attached to e-cigarettes in prisons. These appeared similar to those previously caused by tobacco prior to smoke-free prison policy and are to be expected, given prison rules on purchasing items from the prison shop and the informal economy in prisons.(30) (Analysis of canteen spend will be reported separately.) Nonetheless, some staff might view such issues as particularly troublesome at a time when the workforce is facing wider challenges including increasing numbers of PiC, staff sickness absence rates and increased working hours.(31)

Finally, staff reported potential limitations or challenges of e-cigarettes for reducing tobacco harms in PiC long-term. Discomfort with continued use of nicotine among PiC may stem from staff knowledge that some individuals have a strong desire to become 'free' of substance dependence, as well as from (mis)understandings of health risks of nicotine (32) or

For peer review only - http://bmjopen.bmj.com/site/about/guidelines.xhtml

perceptions that someone who vapes has not truly stopped 'smoking'.(33) Staff members' acknowledgement of risks that many PiC might resume smoking soon after leaving smoke-free prisons, despite having exclusively vaped for a period, reflects the substantial challenges that people often face in transitioning from prison to the outside world.(34) Prison staff did not raise concerns about use of e-cigarettes by the minority of never smokers in the prison population.(35) Ongoing monitoring would be helpful in tracking this issue in the future, so that swift corrective action could be taken if required.

Findings reported here will be of interest to jurisdictions planning on making their prison systems smoke-free and those considering use of e-cigarettes for harm reduction. They highlight the need for any policies on e-cigarette use in prisons to balance the needs of those PiC who wish to use e-cigarettes (in lieu of tobacco) against calls for precautionary measures to limit (or eliminate) e-cigarette vapour exposures because of a lack of long-term evidence on e-cigarette safety. Based on current evidence regarding their effects on air quality and health (36), restricting indoor vaping in prisons to private rooms (cells) would appear proportionate, although there is a need for ongoing monitoring of the evidence and clear communication for the rationale for policies to prison staff. Like smoking previously, where allowed, vaping in prisons is likely to become normative and culturally embedded over time, potentially presenting future challenges for a prison service should the need to introduce tighter restrictions on e-cigarette use arise. Other challenges associated with the use of e-cigarettes among PiC might be reduced through providing guidance and support on appropriate use and on how to limit or quit use if desired. New practitioner guidance has been developed in Scotland subsequent to these staff interviews, to support PiC to reduce or cease use of e-cigarettes (37) and smoking relapse prevention interventions spanning the pre-post release period. Further research is planned to assess use and impact of this guidance, and to understand whether further measures are required to support behaviour change (e.g. changes

For peer review only - http://bmjopen.bmj.com/site/about/guidelines.xhtml

to the e-cigarettes that are sold in prison). Broader responses are also required to tackle illicit drug use among PiC, including measures to reduce environmental drivers of prison drug use.(38)

We believe the use of one-to-one interviews in this study is a strength because they enable in-depth exploration of people's perspectives and of confidential or sensitive issues (39). Findings from this study complement and extend our previous work exploring staff perspectives on e-cigarettes using data gathered via focus groups before announcement of the smoke-free policy,(13) and again post implementation.(9) A key study limitation is that certain participant groups (e.g. females, current vapers) may not be adequately represented in the sample, limiting our ability to explore potential between-group differences in perspectives. However, the inclusion of staff from a diverse set of prisons provides confidence that the breadth of challenges associated with e-cigarettes in prisons are likely to be reflected well in the data. It is important to note that data were collected prior to the introduction of guidance for practitioners on how to support PiC to reduce or stop using e-cigarettes and before the COVID-19 pandemic, both of which may have important implications for vaping in prisons and are priorities for future studies.

In conclusion, this detailed analysis, focused on the *challenges* associated with vaping in prisons from the perspective of prison staff, identified five main issues: staff concerns about exposures to e-cigarette vapour at work; use of e-cigarettes for illicit drug taking among PiC; the value attached to, and trading of, e-cigarette products in prisons; and implications for long-term nicotine and tobacco use. Addressing these challenges is likely to require a combination of measures specific to e-cigarettes and broader measures to address (illicit) drug use and promote overall health.

Acknowledgements

For peer review only

We are grateful to the prison staff who took part in the interviews, staff within the Scottish Prison Service who assisted with the study and facilitated access, and NHS staff who have supported our research on smoke-free prisons.

Contributors

KH, AB, HS and LB developed the study, with input from RO, DE, RP and the SPS Research Advisory Group. AB and KH liaised with the prison service and reported back on ongoing findings. AB prepared the ethics applications. RO, AB and DE conducted the qualitative interviews; all are highly experienced qualitative researchers. AB, RO and DM conducted analysis using the Framework approach. RO, AB and KH drafted the manuscript and all revisions. All other authors read a subsample of the transcripts and contributed to interpretation of the data and review of the final manuscript.

Ethical approval

Ethical approval was granted by the SPS Research Access and Ethics Committee (approval confirmed 23.8.18; no ref number) and by the University of Stirling General University Ethics Panel (ref: GUEP 497).

Data availability statement

No additional data available.

For peer review only

References

1 Brown A, O'Donnell R, Eadie D, *et al.* Initial Views and Experiences of Vaping in Prisons: A Qualitative Study With People in Custody Preparing for the Imminent Implementation of Scotland's Prison Smokefree Policy. *Nicotine Tob Res* 2021;**23**:543-549.

2 NHS Health Scotland. Smoke-free prisons pathway: A service specification supporting people in our care. 2018. http://www.healthscotland.scot/media/2276/smoke-free-prisons-pathway-a-service-specification-supporting-people-in-our-care.pdf (Accessed 24 September 2021).

3 Woodall J, Tattersfield A. Perspectives on implementing smoke-free prison policies in England and Wales. *Health Promot Int* 2018;**33**):1066-1073.

4 Kennedy SM, Davis SP, Thorne SL. Smoke-free policies in U.S. Prisons and jails: A review of the literature. *Nicotine Tob Res* 2015;**17**:629-35.

5 Sweeting H, Demou E, Brown A, *et al.* Prisoners and prison staff express increased support for prison smoking bans following implementation across Scotland: results from the Tobacco In Prisons study. *Tob Control* 2020;**30**:597-598.

6 Brown A, O'Donnell R, Eadie D, *et al.* E-cigarette Use in Prisons With Recently Established Smokefree Policies: A Qualitative Interview Study With People in Custody in Scotland. *Nicotine Tob Res* 2020;23:939-946.

7 Brown A, Sweeting H, Logan G, *et al.* Prison Staff and Prisoner Views on a Prison Smoking Ban: Evidence From the Tobacco in Prisons Study. *Nicotine Tob Res* 2018;**21**:1027-1035.

8 Scottish Prison Service. Creating a smoke free prison environment: a summary of responses to the public consultation exercise. 2017. http://www.sps.gov.uk/nmsruntime/saveasdialog.aspx?fileName=Public+Consultation+Response+Summary5382_2497.pdf (Accessed 13 December 2021)

9 Brown A, Mitchell D, Hunt K. Post-implementation perspectives on smokefree prison policy: a qualitative study with staff and people in custody. *Eur J Public Health* Published Online First: 27 August 2021. doi:10.1093/eurpub/ckab075

10 Beard E, West R, Michie S, *et al.* Association between electronic cigarette use and changes in quit attempts, success of quit attempts, use of smoking cessation pharmacotherapy, and use of stop smoking services in England: time series analysis of population trends. *BMJ* 2016;**354**:i4645.

11 Hartmann-Boyce J, McRobbie H, Lindson N, *et al.* Electronic cigarettes for smoking cessation. *Cochrane Database of Systematic Reviews* 2020;**10**:CD010216. doi:10.1002/14651858.CD010216.pub4

12 National Academies of Sciences Engineering and Medicine. Public Health Consequences of E-Cigarettes. Conclusions by Outcome. 2018. https://www.nap.edu/resource/24952/012318ecigaretteConclusionsbyOutcome.pdf

13 Brown A, Sweeting H, Semple S, *et al.* Views of prison staff in Scotland on the potential benefits and risks of e-cigarettes in smoke-free prisons: a qualitative focus group study. *BMJ Open* 2019;**9**:e027799.

14 Curry L, Lee YO, Rogers T. E-cigarettes made especially for inmates. *Tob Control* 2014;**23**:e87-e8.

15 Young-Wolff KC, Karan LD, Prochaska JJ. Electronic cigarettes in jails: a panacea or public health problem? *JAMA Psychiatry* 2015;**72**:103-104.

16 Brown A, O'Donnell R, Eadie D, *et al.* E-cigarette Use in Prisons With Recently Established Smokefree Policies: A Qualitative Interview Study With People in Custody in Scotland. *Nicotine Tob Res* 2021;**23**:939-46.

17 Spencer L, Ritchie J, O'Connor W, Morrell G, Ormston R. Analysis in Practice. In: Ritchie J, Lewis J, McNaughton Nicholls C, Ormston R, eds. Qualitative Research Practice. Sage; London;2014: 295-343.

18 Gale NK, Shapiro J, McLeod HS, *et al.* Patients-people-place: developing a framework for researching organizational culture during health service redesign and change. *Implement Sci* 2014;**9**:106.

For peer review only - http://bmjopen.bmj.com/site/about/guidelines.xhtml

19 Kinman G, Clements AJ, Hart J. Job demands, resources and mental health in UK prison officers. *Occup Med* 2017;**67**:456-460.
20 McLean J, Dean L. Scottish Health Survey 2018: Main report - Revised 2020.
21 Scottish Prison Service. 17th Prisoner Survery 2019. http://www.sps.gov.uk/Corporate/Publications/Publication-7196.aspx (Accessed 13 December 2021).
22 Blackwell AKM, Kosīte D, Marteau TM, *et al.* Policies for Tobacco and E-Cigarette Use: A Survey of All Higher Education Institutions and NHS Trusts in England. *Nicotine Tob Res* 2020;**22**:1235-1238.
23 Stepney M, Aveyard P, Begh R. GPs' and nurses' perceptions of electronic cigarettes in England: a qualitative interview study. *Br J Gen Pract* 2019;**69**:e8-e14.
24 Mello S, Bigman CA, Sanders-Jackson A, *et al.* Perceived Harm of Secondhand Electronic Cigarette Vapors and Policy Support to Restrict Public Vaping: Results From a National Survey of US Adults. *Nicotine Tob Res* 2016;**18**:686-693.
25 Kolind T, Duke K. Drugs in prisons: Exploring use, control, treatment and policy. *Drugs Educ Prev Pol* 2016;**23**:89-92.
26 Ralphs R, Williams L, Askew R, *et al.* The development of a synthetic cannabinoid market in an English prison. *Int J Drug Policy* 2017;**40**:57-69.
27 Corazza O, Coloccini S, Marrinan S, *et al.* Novel Psychoactive Substances in Custodial Settings: A Mixed Method Investigation on the Experiences of People in Prison and Professionals Working With Them. *Front Psychiatry* 2020;11:460.
28 Gooch K, Treadwell J. Prisoner Society in an Era of Psychoactive Substances, Organized Crime, New Drug Markets and Austerity. *B J Criminol* 2020;**60**:1260-1281.
29 Breitbarth AK, Morgan J, Jones AL. E-cigarettes—An unintended illicit drug delivery system. *Drug Alcohol Depend* 2018;1**92**:98-111.
30 Walker DF. The informal economy in prison. *Crim Justice Matters*. 2015;**99**:18-19.
31 Auditor General. The 2018/19 audit of the Scottish Prison Service. 2020. https://www.audit-scotland.gov.uk/uploads/docs/report/2019/s22_190912_sps.pdf (Accessed 24 September 2021).
32 O'Brien EK, Nguyen AB, Persoskie A, *et al.* U.S. adults' addiction and harm beliefs about nicotine and low nicotine cigarettes. *Prev Med* 2017;**96**:94-100.
33 Hartwell G, Egan M, Petticrew M. Understanding decisions to use e-cigarettes or behavioural support to quit tobacco: a qualitative study of current and ex-smokers and stop smoking service staff. *Addiction* 2020;**115**:518-526.
34 Puljevic C, Segan CJ. Systematic Review of Factors Influencing Smoking Following Release From Smoke-Free Prisons. *Nicotine Tob Res* 2019;**21**:1011-20.
35 Scottish Prison Service. 16th Prisoner Survey. 2017. https://www.sps.gov.uk/Corporate/Publications/Publication-6101.aspx (Accessed 13 December 2021)
36 Li L, Lin Y, Xia T, *et al.* Effects of Electronic Cigarettes on Indoor Air Quality and Health. *Annu Rev Public Health* 2020;**41**:363-80.
37 NHS Health Scotland. Supporting individuals to cut down and quit vaping: Guidance for NHS Quit Your Way advisers based in prisons. 2020. http://www.healthscotland.scot/media/2955/supporting-individuals-to-cut-down-and-quit-vaping.pdf (Accessed 24 September 2021)
38 Mjåland K. Exploring prison drug use in the context of prison-based drug rehabilitation. *Drugs Educ Prev Pol* 2016;**23**:154-62.
39 Guest G, Namey, E. & Mitchell, M. In-depth interviews. *Collecting Qualitative Data*. SAGE Publications, Ltd. 2013:113-171.

For peer review only - http://bmjopen.bmj.com/site/about/guidelines.xhtml

COREQ (COnsolidated criteria for REporting Qualitative research) Checklist

A checklist of items that should be included in reports of qualitative research. You must report the page number in your manuscript where you consider each of the items listed in this checklist. If you have not included this information, either revise your manuscript accordingly before submitting or note N/A.

Topic	Item No.	Guide Questions/Description	Reported on Page No.
**Domain 1: Research team and reflexivity**			
Personal characteristics			
Interviewer/facilitator	1	Which author/s conducted the interview or focus group?	
Credentials	2	What were the researcher's credentials? E.g. PhD, MD	
Occupation	3	What was their occupation at the time of the study?	
Gender	4	Was the researcher male or female?	
Experience and training	5	What experience or training did the researcher have?	
Relationship with participants			
Relationship established	6	Was a relationship established prior to study commencement?	
Participant knowledge of the interviewer	7	What did the participants know about the researcher? e.g. personal goals, reasons for doing the research	
Interviewer characteristics	8	What characteristics were reported about the inter viewer/facilitator? e.g. Bias, assumptions, reasons and interests in the research topic	
**Domain 2: Study design**			
Theoretical framework			
Methodological orientation and Theory	9	What methodological orientation was stated to underpin the study? e.g. grounded theory, discourse analysis, ethnography, phenomenology, content analysis	
Participant selection			
Sampling	10	How were participants selected? e.g. purposive, convenience, consecutive, snowball	
Method of approach	11	How were participants approached? e.g. face-to-face, telephone, mail, email	
Sample size	12	How many participants were in the study?	
Non-participation	13	How many people refused to participate or dropped out? Reasons?	
Setting			
Setting of data collection	14	Where was the data collected? e.g. home, clinic, workplace	
Presence of non-participants	15	Was anyone else present besides the participants and researchers?	
Description of sample	16	What are the important characteristics of the sample? e.g. demographic data, date	
Data collection			
Interview guide	17	Were questions, prompts, guides provided by the authors? Was it pilot tested?	
Repeat interviews	18	Were repeat inter views carried out? If yes, how many?	
Audio/visual recording	19	Did the research use audio or visual recording to collect the data?	
Field notes	20	Were field notes made during and/or after the inter view or focus group?	
Duration	21	What was the duration of the inter views or focus group?	
Data saturation	22	Was data saturation discussed?	
Transcripts returned	23	Were transcripts returned to participants for comment and/or	

For peer review only

Topic	Item No.	Guide Questions/Description	Reported on Page No.
		correction?	
**Domain 3: analysis and findings**			
Data analysis			
Number of data coders	24	How many data coders coded the data?	
Description of the coding tree	25	Did authors provide a description of the coding tree?	
Derivation of themes	26	Were themes identified in advance or derived from the data?	
Software	27	What software, if applicable, was used to manage the data?	
Participant checking	28	Did participants provide feedback on the findings?	
Reporting			
Quotations presented	29	Were participant quotations presented to illustrate the themes/findings? Was each quotation identified? e.g. participant number	
Data and findings consistent	30	Was there consistency between the data presented and the findings?	
Clarity of major themes	31	Were major themes clearly presented in the findings?	
Clarity of minor themes	32	Is there a description of diverse cases or discussion of minor themes?	

Developed from: Tong A, Sainsbury P, Craig J. Consolidated criteria for reporting qualitative research (COREQ): a 32-item checklist for interviews and focus groups. *International Journal for Quality in Health Care*. 2007. Volume 19, Number 6: pp. 349 – 357

Once you have completed this checklist, please save a copy and upload it as part of your submission. DO NOT include this checklist as part of the main manuscript document. It must be uploaded as a separate file.

For peer review only
